# Integrating field surveys and remote sensing data to study distribution, habitat use and conservation status of the herpetofauna of the Comoro Islands

**DOI:** 10.3897/zookeys.144.1648

**Published:** 2011-11-03

**Authors:** Oliver Hawlitschek, Boris Brückmann, Johannes Berger, Katie Green, Frank Glaw

**Affiliations:** 1 Zoologische Staatssammlung München (Bavarian State Collection of Zoology), Münchhausenstr. 21, 81247 München, Germany; 2 Bristol Conservation and Science Foundation, Comoros Project Office, Anjouan, Union des Comores

**Keywords:** amphibians, area of occupancy, conservation planning, IUCN Red List, Landsat, protected areas, reptiles, satellite imagery, Western Indian Ocean

## Abstract

We studied the non-marine reptile and amphibian species of the volcanic Comoro archipelago in the Western Indian Ocean, a poorly known island herpetofauna comprising numerous microendemic species of potentially high extinction risk and widespread, non-endemic and often invasive taxa. According to our data, the Comoro islands are inhabited by two amphibian species and at least 28 species of reptiles although ongoing genetic studies and unconfirmed historical records suggest an even higher species diversity. 14 of the 28 currently recognized species of terrestrial reptiles (50%) and the two amphibians are endemic to a single island or to the Comoro archipelago. The majority of species are most abundant at low elevation. However, a few endemic species, like the gekkonid lizards *Paroedura sanctijohannis* and *Phelsuma nigristriata*, are more common in or even confined to higher altitudes. We created habitat maps from remotely sensed data in combination with detailed species distribution maps produced using comprehensive data from field surveys between 2000 and 2010, literature, and historical locality records based on specimens in zoological collections. Using these data, we assessed the conservation status of the endemic terrestrial reptiles and amphibians according to the IUCN Red List criteria. Our results show that although little area of natural forest remains on the Comoros, many species are abundant in degraded forest or plantations. Competition and predation by invasive species appears to be the most important threat factor for the endemic herpetofauna, together with habitat degradation and destruction, which further favours invasive species. We propose the status Endangered for three species, Vulnerable for one species, Near Threatened for six species, Least Concern for four and Data Deficient for two species. The endemic subspecies *Oplurus cuvieri comorensis* is proposed for the status Critically Endangered. Based on the results of this study, seven areas of importance for reptile and amphibian conservation on the Comoros are identified. This study shows how remote sensing data can contribute to increasing accuracy and objectiveness of conservation assessments.

## Abstract (fr)

Nous avons étudié les reptiles et amphibiens non marinsde l´archipel volcanique des Comores, Océan Indien occidental, une herpétofaune insulaire peu connue et comprenante à la fois de nombreuses espèces microendémiques à risque d´extinction potentiellement élevé, et des espèces non endémiques à grande aire de distribution, souvent invasives. D´après nos données, l´archipel des Comores est habité par 2 espèces d´amphibiens et au moins 28 espèces de reptiles, bien que des études génétiques en cours et des données historiques non confirmées suggèrent une diversité spécifique plus élevée. 14 des 28 espèces de reptiles terrestres actuellement référencés (50%) et les 2 espèces d´amphibiens sont endémiques d´une seule île ou de l´archipel. La majorité des espèces sont plus abondantes à basse altitude. Cependant quelques espèces endémiques comme les lezards Gekkonidae *Paroedura sanctijohannis* et *Phelsuma nigristriata* sontplus communs ouconfinés à l'altitude élevée. Nous avons réalisé des cartes des habitats à partir de données de télédétection ainsi que des cartes détaillées de distribution des espèces en combinant des données obtenues au cours de mission de terrain entre 2000 et 2010, des données de la littérature et des données de distribution historiques basées sur des spécimens de collections zoologiques. A partir de ces données, nous avons évalué le statut de conservation des espèces endémiques de reptiles terrestres et d´amphibiens d´après les critères de la liste rouge de l´UICN. Nos resultats montrent que bien qu´il reste de petites zones de forêt naturelle aux Comores, beaucoup d´espèces sont abondantes dans les forêts degradés et les plantations. La competition et la prédation par les espèces invasives apparaît comme la plus grosse menace pesant sur l´herpétofaune endémique, aux quelles il convient d´ajouter les dégradations et la destruction des habitats, qui en outré favorise les espèces invasives. Nous proposons le statut Endangered (en danger) pour 3 espèces, Vulnerable (vulnérable) pour une espèce, Near Threatened (quasi menacée) pour 6 espèces, Least Concern (preoccupation mineure) pour 4 et Data Deficient (données insuffisantes) pour 2 espèces. Le statut Critically Endangered (danger critique d’extinction) est proposé pour la sous-espèce *Oplurus cuvieri comorensis*. Sur la base de ces résultats, 7 régions d´importance pour la conservation des reptiles et amphibiens des Comores sont identifiées. Cette étude montre comment les données de télédétection peuvent contribuer à accroître la précision et l’objectivité des estimations de conservation.

## Introduction

Island faunas are considered especially vulnerable to threats and extinction because of the small areas occupied by species and the inability to compensate losses resulting from environmental influences (Diamond 1984a, 1984b, Purvis et al. 2000, Cole et al. 2005, Rödder et al. 2010, Lee and Jetz 2011). High extinction rates on islands have been documented for birds (Temple 1985) and reptiles (Case et al. 1992, Cheke and Hume 2008).

In order to predict and prevent future extinctions, it is necessary to detect threatened species and assess the degree of threat they are facing. The IUCN Red List has become the reference to the status of threatened species worldwide and an important tool in biodiversity monitoring and conservation planning (Stuart et al. 2004, Rodrigues et al. 2006, Vié et al. 2008, Hoffmann et al. 2010). Initially, Red List status assessments were based solely on the experience and opinion of the assessors; no formal criteria were available (Burton 2003). The first set of objective criteria was released in IUCN (1994), and revised in IUCN (2001a), to be henceforth applied in Red List assessments. These criteria are either based on known numbers of individuals or areas inhabited by the species in question. While numbers of individuals may be reliably estimated or counted in large, conspicuous and well-known species, this is often difficult if species are small, elusive or occur in remote areas. In such cases, the criteria based on area are more useful. Buchanan et al. (2008) used remote sensing data to estimate deforestation and its impact on the conservation status of birds in New Britain. To date we know of no other attempts to directly link remote sensing information with IUCN Red List assessments. We suggest that, in cases where sufficient distributional data are available, remote sensing may provide an important contribution towards the objectification of Red List assessments.

For a case study, we chose the Comoro archipelago in the Western Indian Ocean (WIO). The WIO is a region in which particularly many extinction events of reptiles have occurred. Half of the 20 reptile species listed as extinct in the IUCN Red List were endemic to this area (IUCN Red List of Threatened Species, Version 2010.4, http://www.iucnredlist.org; downloaded on 27 October 2010). Due to the relative scarcity of historical records and the absence of identifiable fossil and subfossil material (but see Gerlach and Canning 2004 for a note on possible fragments of giant tortoises), no reliable hypotheses on possible past extinctions on the Comoros can be made. Similarly, no information on possible threats to their endemic extant herpetofauna has been available so far.

The Comoro archipelago consists of four major islands: Grand Comoro (1010 sqkm), Anjouan (424 sqkm), Mohéli (217 sqkm) and Mayotte (377 sqkm). They are a result of hot-spot volcanism (Emerick and Duncan 1982) and were not connected to each other or any other landmasses at any point in their geological history (Colonna et al. 1996, Battistini and Verin 1984). Distances between the islands are from 50 to 90 kilometres and roughly 300 kilometres both to the East African coast and to Madagascar. According to various authors the archipelago’s oldest island, Mayotte, has an estimated age of between 15 and 3.65 ma (million years) (Hajash and Armstrong 1972, Montaggioni and Nougier 1981, Emerick and Duncan 1982, Nougier et al. 1986, Bachélery and Coudray 1993). Climate on the Comoros is tropical and dominated by monsoon and trade winds.

Seven families of terrestrial reptiles and one family of amphibians are recorded on the Comoros. The highest species diversity is found in the geckos (14 species), followed by skinks (4 species), lamprophiid (3 species) and typhlopid snakes (3 species), chameleons (2 species), one species of iguana (*Oplurus cuvieri comorensis*), and one species of agamid lizard (*Agama agama*). Two species of amphibians are endemic, but still await description (Vences et al. 2003). Sea turtles commonly use Comorian beaches for reproduction but are not included in this study as we focus on the terrestrial herpetofauna. The Nile Crocodile (*Crocodylus niloticus*) was reported to occur on the Comoros by Benson (1960), but has not been observed since. Benson’s report probably refers to an erratic specimen (Meirte 2004).

The iguana *Oplurus cuvieri comorensis* is endemic to Grand Comoro at subspecies level, the nominate form inhabiting Madagascar (Münchenberg et al. 2008). Each of the four subspecies of *Cryptoblepharus boutonii* (Scincidae) is endemic to one of the four islands, while some other subspecies of the same species are distributed over a vast area in the Indian and Pacific oceans (Rocha et al. 2006). *Phelsuma v-nigra* (Gekkonidae) is endemic to the Comoros as a species, but Grand Comoro, Mohéli and Anjouan each have one endemic subspecies (Rocha et al. 2009a).

As in many regions worldwide, natural habitats on the Comoros are threatened by the impact of man. The archipelago was first colonized by man over a thousand years ago (Allibert and Verin 1996, Cheke 2010, Msaidie et al. 2011). Today, the Comoros are densely populated islands; calculated from numbers of various sources (Central Intelligence Agency 2010, INSEE 2007), densities are about 325 inhabitants per sqkm for Grand Comoro, 595 for Anjouan, 167 for Moheli and 533 for Mayotte. In comparison, the numbers are 117 inhabitats per sqkm for metropolitan France and 404 for the Netherlands, Europe’s most densely populated country. An estimated growth rate of 2.7% makes the Comorian population one of the fastest growing on earth. Due to requirements for construction, ylang ylang distillation, and agriculture, most forests in easily accessible areas are lost, and the remaining forests are already modified by activities of logging and agroforestry (Anonymus 2000, Kothari 2003). Jolly and Fukuda-Parr (2000) calculated an average deforestation rate of 5.8% per year for the period from 1990 to 1995, which is the fourth highest rate worldwide.

Due to increasing sea and air traffic, foreign species are regularly introduced and sometimes become invasive. Most prominent among the invasive plants is the strawberry guava (*Psidium littorale*) on Grand Comoro, which has already had a devastating impact on native floras on Mauritius and Hawaii (Lowe et al. 2000, Tassin et al. 2006). Many animal species, among them rats, were already introduced in historical times (Allibert et al. 1983, Atkinson 1985). Four species of *Hemidactylus* house geckos inhabit the Comoros, all of which were probably introduced historically or recently (Vences et al. 2004, Rocha et al. 2005). Several *Hemidactylus* species including those occurring on the Comoro archipelago are considered invasive in many tropical areas and *Hemidactylus frenatus* has probably led to the extinction of the endemic species of *Nactus* geckos on the Mascarene Islands (Cole et al. 2005). A case of very recent introduction is the agamid lizard *Agama agama*. According to Meirte (2004) this species was introduced to Grand Comoro in 1998, and already in early 2000 it was found to be abundant in parts of the capital Moroni (Wagner et al. 2009).

In this paper, we present distribution maps for all terrestrial species of Comorian reptiles and amphibians. Furthermore, we present the first complete terrestrial habitat survey based on satellite imagery for the Comoros archipelago, which we utilised to evaluate which habitats are of importance for the herpetological species. We also present a key to the reptiles and amphibians of the Comoro Islands to facilitate future identification. Based on these results, we use IUCN Red List criteria to assess the conservation status of the Comorian reptiles and amphibians and propose areas of particular conservational importance.

## Methods

### Locality data and species identification

Locality data on reptiles and amphibians was obtained from zoological collections, field surveys, and literature. We surveyed all preserved Comorian specimens of terrestrial reptiles and amphibians from the following collections: Natural History Museum, London, United Kingdom (BMNH); Muséum National d’Histoire Naturélle, Paris, France (MNHN); Senckenberg Forschungsinstitut und Naturkundemuseum Frankfurt, Germany (SMF); Zoologisches Forschungsmuseum Alexander Koenig, Bonn, Germany (ZFMK); Museum für Naturkunde Berlin, Germany (ZMB); Zoologische Staatssammlung München, Germany (ZSM). In addition we discuss further records of special interest fom the California Academy of Sciences, San Francisco (CAS) and the Field Museum, Chicago (FMNH) which were traced by searches in the HerpNET database (http://herpnet.org). Material from field surveys and museums were also used for creation of the identification key.

Only reliable localities of safely identified specimens have been included in this study. All recent locality data was collected in repeated field surveys in the Comoros in^ 2000^, 2002, 2008 and 2010, with a total of 24 weeks of field work or obtained from Carretero et al. (2005). We also visited historical localities in an attempt to confirm the presence of the species historically recorded. To verify species identifications, voucher specimens (deposited in ZSM) and / or tissue samples were collected and photographs were taken. In the field, coordinates were stored with a Garmin (R) ETREX Venture GPS.

### Habitat classes

During the herpetological surveys, we also collected ground truthing data for habitat mapping. Ten habitat classes were selected based on their different suitability as habitats for reptiles and amphibians, and ease of identification both in the field and in remote sensing applications. Where appropriate, classes were selected in accordance with the IUCN habitats authority file (IUCN 2001b), and classification numbers of this file are given in brackets. Basic information on Comorian vegetation was obtained from Anonymus (2000), Pascal (2002) and Louette et al. (2004).

**Closed Forest:** Forest with little or no traces of human impact and a closed canopy. The remote sensing classification does not allow the discrimination between areas of undisturbed pristine forest and forest which has a closed canopy but is modified by low intensity human activities such as limited agro-foresty, selective logging, and firewood collecting. However, the methods applied here have already been used to discriminate areas of forest under heavier human influence (Hayes and Sader 2001, Sader et al. 2001, Wilson and Sader 2002). Corresponsing classes in IUCN (2001b) are Tropical Moist Lowland (1.6) and Tropical Moist Montane Forests (1.9)

**Mangrove:** TropicalMangrove Forests (1.7), dominated by *Rhizophora* and *Avicennia* trees, growing in estuaries and coastal areas.

**Montane Dry Vegetation:** Tropical High Altitude Shrubland (3.7), dominated by *Philippia (Erica) comorensis*. Restricted to Mt. Karthala on Grand Comoro, at altitudes of above 1400 meters.

**Volcanic Rock:** Areas covered by recent lava outflow. Vegetation is absent or very scarce. Also includes large cliff and shore areas of volcanic rock.

**Inland Water:** Water bodies within the terrestrial surface of the islands that have no connection to the sea. This includes freshwater (e.g. Lac Dziani Dzaha) as well as other types of water (e.g. Lac Salé).

**Degraded Forest:** Tropical Heavily Degraded Former Forest (11.6) in forest areas where logging, intensive agroforestry, and clearing for small plantations are present. It also includes regenerating secondary forests. This habitat is distinguished from natural forest types by a more open canopy and thus less reflectance in the near-IR bands in relation to reflectance in the visible spectrum. Patches of crop plants are present, either under the original canopy or in clearings. Neophyte stands are common.

**Plantation:** Areas widely dominated by plantations of arboreal and arbusteal crops (11.3). Only isolated stands, single trees or nothing is left of the primary forest. In relation to forest and degraded forest, near-IR reflectance is lower. This class does not include pure open crop plantations (manioc, potato etc.). On the Comoros, however, such crops are rarely planted in sufficiently large pure plantations to justify a separate class. Patches of open crop plantations may be included in the classes “Plantation” or “Dry / Low Vegetation”.

**Dry / Low Vegetation:** Characterized by complete or near complete vegetation cover in dry areas. It is dominated by shrubby or herbaceous plants (Tropical Dry Shrubland: 3.5), but may also contain stands of short trees or single larger trees, often forming savanna-like structures (Dry Savanna: 2.1). Baobabs (*Adansonia digitata*) often occur in this class. This class also includes plantations in dry areas, which are often to a high degree mixed with natural vegetation (11.3). Near-IR reflectance is lower than in other vegetation classes because of relatively low photosynthetic activity due to drought.

**Openly Populated Area:** Urban areas (11.5) with gardens (Rural Gardens: 11.4), small plantations, and trees which often shade buildings. More than 50% vegetation cover.

**Densely Populated Area:** Denser urban areas (11.5), dominated by buildings and infrastructure, with less than 50% vegetation cover.

### Habitat and distribution maps

We created habitat maps of all four Comoro islands based on Landsat ETM+ images of 30 m resolution, purchased from Landsat.org, Global Observatory for Ecosystem Services, Michigan State University (http://landsat.org, 2007-09-20; images et1610690228032, et1620680814032, et1620690520012, and et1630681114052, years 2001 to 2007). Elevation data was obtained from SRTM-DEM images of 90 m resolution from the information technology center at the University of Tokyo (http://itc.u-tokyo.ac.jp/GIS, 2007-10-10; images S13E044, S12E043, S13E043, and S13E045).

Habitat mapping was conducted using the maximum likelihood supervised classification in ENVI 4.3 to 4.7, ITT Industries, Inc., followed by manual processing in ESRI ArcGIS 9.2 and 9.3. We based our supervised classification on ground verification points collected in the field, using 10% to 25% of the available field points as test points instead of training points. Further accuracy assessment of the classification was conducted by visual comparison of habitat classes between the classified map, topographic maps (1:25,000 for Mayotte and 1:50,000 for all other islands, © Institut Géographique National, France, 1993 and 1995) and GoogleEarth scenes (http://earth.google.com/). We then used ArcGIS 9.2 to 10.0 to measure the areas covered by the different habitat classes and to create distribution maps for all species.

### Conservation assessments

We evaluated all endemic species of reptiles and amphibians in the Comoros using the IUCN Red List criteria (IUCN 2001a). These assessments are considered proposals for a national conservation status as well as IUCN Red List status and are currently under review at the CI-IUCN Biodiversity Assessment Unit. To allow an objective application of this guideline, we calculated extent of occurrence (area potentially inhabitable by a species) and area of occupancy (part of this area in which a species actually lives; see IUCN 2001a for further explanation) based on our habitat classification. Historical records which could not be confirmed by surveys after 1999 were not included in these calculations, because the habitat class of these localities may have changed between the time of recording and today. Unless a species was known to be restricted to a certain part of an island, we considered the total area of all islands inhabited by a species as this species’ extent of occurrence. To calculate the area of occupancy, we subtracted all area that was outside the species’ altitudinal range or that was covered by habitat classes in which the species was not recorded from the extent of occurrence. For certain species, habitat classes were excluded from the area of occupancy even if specimens had been observed there because they were considered unsuitable for maintaining a viable population, as explained for each of these species in the discussion. Conservation assessments and proposals of IUCN Red List status are presented under Discussion: Conservation assessments.

## Results

### Distribution maps and species composition

We examined 680 historical specimens in herpetological collections which are known to harbour significant material from the Comoro islands (BMNH, MNHN, SMF, ZFMK and ZMB), representing 306 localities recorded between 1845 and 1991. Of all historical localities, 56 had very imprecise locality data (e.g., “Comoros”). 205 were referable to one particular island of the archipelago. Only 45 had locality data precise enough to be used for our distribution maps, and all but two of these could be confirmed in our field surveys. Thus, these rather precise historical records provided very valuable support in our surveys. In our field surveys from 2000 to 2010, we collected 311 specimens and recorded 952 localities. We obtained another 133 locality records collected in 2003 from Carretero et al. (2005). After exclusion of overlapping records, a total of 1038 localities were used in our analyses and for the creation of the distribution maps (Appendix 1, 2).

Our data (Table 1) show that seven families of reptiles are present on the Comoros. 14 of the 28 recognized species of terrestrial reptiles (50%) and both amphibian species (100%) are endemic to a single island or to the Comoros. Of the 36 taxa of both groups recognized at species or subspecies level, 22 (66.7%) are endemic. Within the Comoro archipelago amphibians have only been recorded on Mayotte, where the two species are endemic, but to date undescribed. One species of blindsnake (*Typhlops* sp.) is probably undescribed. Photographs of the species and subspecies are shown in Figs 5–9.

**Table 1.** An overview of species and subspecies of Comorian reptiles and amphibians. EI = Endemic to this island, EC = Endemic to the Comoros, P = Present on this island, but not endemic.

	*Gd. Comoro*	*Anjouan*	*Mohéli*	*Mayotte*
**Typhlopidae:**				
*Ramphotyphlops braminus*	P	P	P	P
*Typhlops comorensis*	EC	EC	-	-
*Typhlops* sp.	EI	-	-	-
**Lamprophiidae:**				
*Leioheterodon madagascariensis*	P	-	-	-
*Liophidium mayottensis*	-	-	-	EI
*Lycodryas sanctijohannis*	EC	EC	EC	EC
**Chamaeleonidae:**				
*Furcifer cephalolepis*	EI	-	-	-
*Furcifer polleni*	-	EC	-	EC
**Gekkonidae:**				
*Ebenavia inunguis*	P	P	P	P
*Geckolepis maculata*	P	P	P	P
*Hemidactylus frenatus*	P	P	P	P
*Hemidactylus mercatorius*	P	P	P	P
*Hemidactylus parvimaculatus*	-	P	P	-
*Hemidactylus platycephalus*	P	P	P	P
*Paroedura sanctijohannis*	EC	EC	EC	EC
*Phelsuma comorensis*	EI	-	-	-
*Phelsuma dubia*	P	P	P	P
*Phelsuma laticauda*	-	P	-	P
*Phelsuma nigristriata*	-	-	-	EI
*Phelsuma pasteuri*	-	-	-	EI
*Phelsuma robertmertensi*	-	-	-	EI
*Phelsuma v-nigra anjouanensis*	-	EI	-	-
*Phelsuma v-nigra comoraegrandensis*	EI	-	-	-
*Phelsuma v-nigra v-nigra*	-	-	EI	-
**Iguanidae:**				
*Oplurus cuvieri comorensis*	EI	-	-	-
**Scincidae:**				
*Amphiglossus johannae*	EC	EC	EC	EC
*Cryptoblepharus boutonii ater*	EI	-	-	-
*Cryptoblepharus boutonii degrijsii*	-	EI	-	-
*Cryptoblepharus boutonii mayottensis*	-	-	-	EI
*Cryptoblepharus boutonii mohelicus*	-	-	EI	-
*Trachylepis comorensis*	P	P	P	P
*Trachylepis striata*	-	P	-	-
**Agamidae:**				
*Agama agama*	P	-	-	-
**Mantellidae:**				
*Blommersia* sp.	-	-	-	EI
*Boophis* sp.	-	-	-	EI

### Habitat classification

**Figure 1.**
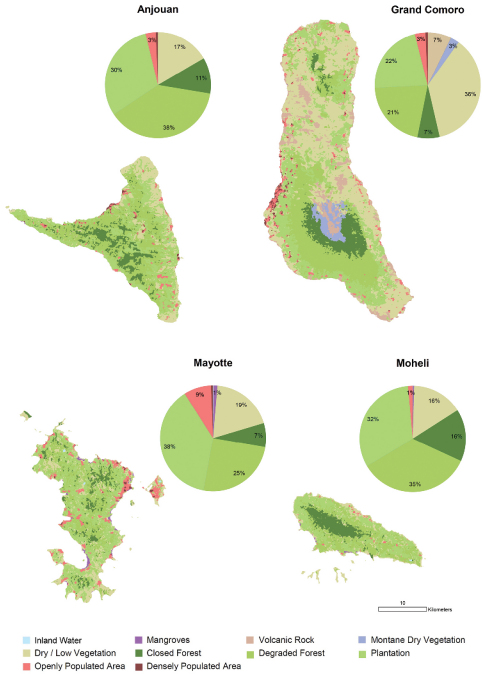
Habitats on the Comoro islands. Maps of habitat classes and relative land cover are given for each island.

**Figure 2.**
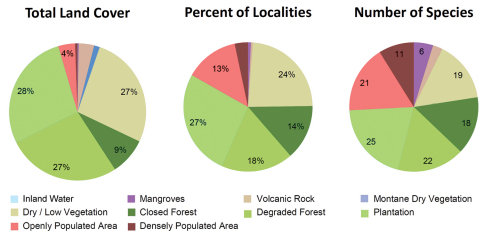
Land cover, localities and number of recorded species

**Figure 3.**
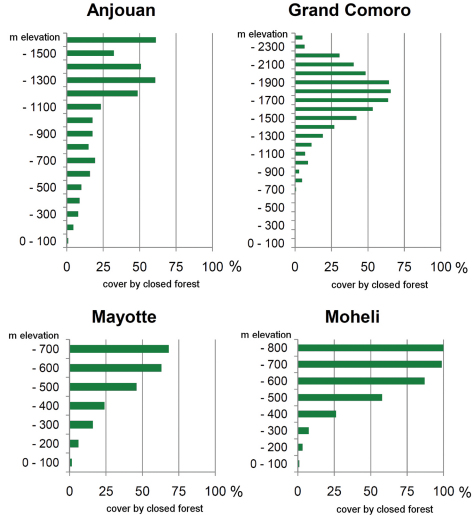
Forest areas on the Comoros. For each level of altitude (in intervals of 100 m), the area occupied by forest is given as percentage of the total area occupied by all habitat classes in this level.

Our habitat classification is supported by a total of 738 ground verification points. Habitat maps are given in Figure 1. The results show that plantation (28%), degraded forest and dry / low vegetation (each 27%) equally contribute to a total of 82% of the land cover of the Comoros (Table 2 and Figure 2). Mayotte is clearly dominated by plantation (38%), Grand Comoro by dry / low vegetation (36%), Anjouan (38%) and Mohéli (35%) by degraded forest. Closed forest makes up only 9% of the total land area of the Comoros (Figure 3). The largest areas remain on Grand Comoro (68.5 sqkm) and cover only 7% of the island’s area. In proportion to island size, the largest area of forest is detected on Mohéli with 16% (34.5 sqkm), followed by Anjouan with 11% (46.6 sqkm). Mayotte displays the smallest remaining area of forest (27.5 sqkm, 7%).

**Table 2.** Land cover by habitat classes. The areas of each habitat class per island and as total are given in square kilometres.

	Anjouan	Grand Comoro	Mayotte	Mohéli	Total
Inland Water	0.00	0.04	0.06	0.02	0.12
Mangroves	0.01	0.07	4.44	0.08	4.60
Volcanic Rock	0.00	73.23	0.00	0.00	73.23
Montane Dry Vegetation	0.00	27.00	0.00	0.00	27.00
Dry / Low Vegetation	70.24	367.95	71.64	33.53	543.37
Closed Forest	46.60	68.48	27.47	34.47	177.02
Degraded Forest	161.27	212.64	95.06	74.84	543.81
Plantation	129.41	220.69	143.50	69.68	563.28
Openly Populated Area	13.22	32.91	31.52	3.03	80.68
Densely Populated Area	2.79	6.85	2.27	0.00	11.91
	**423.54**	**1009.85**	**375.96**	**216.65**	**2025.01**

### Habitat use

Most specimens (27%) and species (25 species) out of the total number of 30 species of reptiles and amphibians were recorded in the habitat class plantation (Figure 2). A similarly high percentage of specimens was recorded in dry / low vegetation (24%), followed by degraded forest (18%). Of specimen records, 14% were made in forest and 13% in openly populated areas. The second highest numbers of species were recorded in degraded forest (22) and in openly populated areas (21), followed by dry and low vegetation (19), forest (18), densely populated areas (11), mangroves (6) and volcanic rock (3).

**Figure 4.**
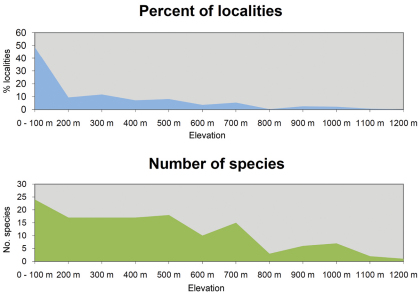
Localities and number of species in relation to altitude.

Regarding altitude, almost half of all localities (48.9%) and 25 species (out of 30) were recorded in altitudes below 100 m asl (Figure 4). The number of species recorded then drops to 18 up to 200 m altitude and still 16 at elevations of up to 700 m. The relative number of records decreases to around 10% up to 200 m and further to a single specimen of *Furcifer cephalolepis* on the slope of Mt. Karthala, Grand Comoro, at 1135 m asl.

Distribution maps and distribution over habitats and altitude levels for all species are given in Figs 10–19.

### Identification key

We created an identification key including all non-marine species and subspecies of amphibians and reptiles on the Comoros. We also included species whose occurrence on the Comoros could not be confirmed. For every species, the islands of its occurrence are indicated in round brackets (AN = Anjouan, GC = Grand Comoro, MA = Mayotte, MO = Mohéli). The key is based on data published in Meirte (2004), Vences et al. (2004), Glaw and Vences (2007) and unpublished data in Hawlitschek (2008) and by F. Glaw and V. Wallach.

**Table:** 

1	Skin moist, no scales present; tail absent	Amphibians / Anurans (frogs), 2
–	Skin dry, covered with scales; tail present	Reptiles, 3
2	Small species (adult snout-vent length 25-35 mm); tympanal region blackish; hind limbs with distinct dark bands; relatively small eyes; iris silvery; adult males with distinct femoral glands on ventral surface of thighs; only on Mayotte	*Blommersia* sp. (MA)
–	Medium-sized species (adult snout-vent length 35-56 mm); tympanal region beige to brown; hind limbs without or with indistinct dark bands; relatively large eyes; iris reddish; adult males without femoral glands on ventral surface of thighs; only on Mayotte	*Boophis* sp. (MA)
3	Limbs and external ear opening absent	Serpentes (snakes), 4
–	Limbs and external ear opening present	Lacertilia (lizards), 11
4	Worm–like snakes with burrowing habits; very small, total length ca. 100-250 mm; scalation and coloration uniform all over body; eyes absent or only visible as small black spots covered by scales	Typhlopidae, 5
–	Typical snakes, ground- or tree-dwelling; medium-sized to large, adult total length > 50 cm; one distinctly enlarged row of ventral shields and 17–23 dorsal scale rows at midbody; pupil of eyes clearly visible	other families, 7
5	Maximum total length 181 mm; 20 midbody scale rows; 261-364 total middorsal scales; superior nasal suture visible dorsally	*Ramphotyphlops braminus* (AN, GC, MA, MO)
–	Not as above	*Typhlops*, 6
6	Maximum total length 245 mm; 22 midbody scale rows; 414-485 total middorsal scales* Typhlops comorensis (AN, GC)*
–	Extremely small, known total length 68-70 mm; 18 midbody scale rows; 384-408 total middorsal scales; species poorly known	*Typhlops* sp. (GC)
7	Dorsal coloration with dark and bright crossbands, dark bands at least twice as broad as bright bands; less than 100 ventral scales; only on Grande Comoro, occurrence in need of confirmation	*Lycodon aulicus* (Natricidae, GC – UNCONFIRMED)
–	Dorsal coloration other than 7 A); more than 150 ventral scales	Lamprophiidae, 8
8	Robust, ground-dwelling and digging snake; rostral scale directed upwards; total length of adults up to 150 cm; 23 dorsal scale rows* Leioheterodon madagascariensis* (GC)
–	Slender, terrestrial or arboreal, non-digging snakes; rostral scale not directed upwards; total length of adults up to 100 cm; 17-19 dorsal scale rows	9
9	Pupil round; head not broader than neck; coloration dorsally grayish, ventrally bright with small dark spots on each ventral scale; ground-dwelling	*Liophidium mayottensis* (MA)
–	Pupil vertical; head distinctly broader than neck; ground- or tree-dwelling	*Lycodryas*, 10
10	Mostly 19 dorsal scale rows (sometimes 17) at midbody; coloration sexually dimorphic, males grayish, sometimes with dark spots or blotches, iris gray, females uniformly orange or brown, iris brownish	*Lycodryas sanctijohannis* (AN, GC, MA, MO)
–	17 dorsal scale rows at midbody, coloration brown with dark crossband	*Lycodryas gaimardii* (MA – UNCONFIRMED)
11	Eyes largely covered with skin and moving independently; prehensile tail; tong-like hands and feet with two or three lateral fingers opposing inner fingers	Chamaeleonidae, 12
–	Eyes not covered with skin; no prehensile tail; fingers and toes not fused	other families, 13
12	Snout-vent length maximum 77 mm in males, 54 mm in females; rostral crests with very distinctly enlarged scales, fusing anteriorly and reaching or exceeding snout tip; distinctly elevated parietal crest; gular crest with distinctly enlarged cones	*Furcifer cephalolepis* (GC)
–	Snout-vent length maximum 104 mm in males, 63 mm in females; rostral crests with weakly enlarged scales, poorly elevated, diminishing posterior to snout tip; parietal crest visible, but not elevated; gular crest poorly developed	*Furcifer polleni* (AN, MA)
13	Head dorsally with enlarged, symmetrical scales distinct from scales following posteriorly	Scincidae / Lacertidae, 14
–	Head dorsally with scales similar to those following posteriorly	other families, 21
14	Dorsal scales granular, ventral scales cycloid and overlapping, forming a distinct collar of enlarged scales in the throat region	*Meroles knoxii* (Lacertidae, AN – UNCONFIRMED)
–	Scales uniformly cycloid and overlapping	Scincidae, 15
15	Body cylindrical, head and tail not clearly distinct from body; limbs small; coloration light brown with dark brown spots	*Amphiglossus johannae* (AN, GC, MA, MO)
–	Body fusiform, distinct neck, tail distincly less broad than body, lizard-like appearance; ground coloration dark brown to black	16
16	Medium-sized, often robust lizards, total length up to more than 200 mm; scale rows around midbody 28 to 39	*Trachylepis*, 17
–	Small lizards, less than 100 mm total length; dorsoventrally depressed; coloration blackish, often with white stripes; scale rows around midbody 21 to 29; only in rocky intertidal regions	*Cryptoblepharus boutonii*, 18
17	Common lizard; coloration dark with patterns of brighter spots; never with white stripes	*Trachylepis comorensis* (AN, GC, MA, MO)
–	Coloration blackish with two white lateral stripes	*Trachylepis striata* (AN)
18	Coloration uniformly black	*Cryptoblepharus boutonii ater* (GC)
–	White stripes present	19
19	Black with two white lines along the body	*Cryptoblepharus boutonii mohelicus* (MO)
–	More than two white lines present	20
20	Black with four white lines along the body, tail sometimes bluish	*Cryptoblepharus boutonii mayottensis* (MA)
–	Black with five white or yellowish lines along the body, tail often blue	*Cryptoblepharus boutonii degrijsiii* (MO)
21	Ventral surface of fingers and toes with adhesive lamellae, allowing movement on vertical surfaces; total length never exceeding 160 mm; usually not ground-dwelling	Gekkonidae, 22
–	Adhesive lamellae absent; total length up to 500 mm; ground-dwelling, climbing only on rough surfaces; robust lizards	other families, 38
22	Dorsal ground color green, sometimes in shades of olive or brownish, often with pattern in red; pupil round; diurnal	*Phelsuma*, 23
–	Dorsal ground color brown or grey, green and red colors absent; pupil vertical; nocturnal	31
23	Dark lateral stripe along body present	24
–	No dark lateral stripe present	25
24	Neck dorsally without dark stripes, limbs dorsally marbled with bright spots, tail dorsally with dark spots	*Phelsuma comorensis* (GC)
–	Neck dorsally with three dark stripes, limbs dorsally not marbled, tail with narrow dark crossbands	*Phelsuma nigristriata* (MA)
25	Dorsal ground color rather green with olive, brown or turquoise shades than deep green	26
–	Dorsal ground color deep green	27
26	Flanks with brighter spots, dorsum dark green or brown, with orange median line (sometimes interrupted), sometimes with blue blotch on neck	*Phelsuma robertmertensi* (MA)
–	No bright spots on flanks, often red dots on dorsum, no median line, tail often bluish	*Phelsuma dubia* (AN, GC, MA, MO)
27	Numerous small yellowish or golden spots dorsally in neck region and on tail; three large red blotches posteriorly on dorsum	*Phelsuma laticauda laticauda* (AN, MA)
–	Neck and tail not yellowish or golden, dorsum with numerous smaller red dots and spots	28
28	Blue spot on neck; gular region uniformly bright	*Phelsuma pasteuri* (MA)
–	No blue color elements dorsally; gular region with more or less visible pattern in the form of a “V”	*Phelsuma v-nigra*, 29
29	Ventral side yellow, dorsal side green with relatively large red dots	*Phelsuma v-nigra v-nigra* (MO)
–	Ventral side whitish, dorsal red dots smaller	30
30	Dorsal side green to bluish, small red dots, sometimes with red median line	*Phelsuma v-nigra comoraegrandensis* (GC)
–	Dorsal side green, red dots very small, sometimes forming a reticulated pattern or absent	*Phelsuma v-nigra anjouanensis* (AN)
31	Scales rather large, cycloid, overlapping, can be shed when touched; tubercles and striped color patterns absent	*Geckolepis maculata* (AN, GC, MA, MO)
–	Scales small, granular, in part tubercular; sometimes with striped color pattern	32
32	Adhesive lamellae covering entire ventral side of digits, in double rows of several pads each; when resting, ventral side of body usually in contact with substrate; colors brown during day, grayish, whitish or pinkish at night	*Hemidactylus / Gehyra*, 33
–	Adhesive lamellae only in two pads near tips of the toes; when resting, ventral side of body usually lifted from substrate; colors always brownish other genera	37
33	Body and tail smooth, no tubercles present; coloration grey, sometimes pinkish	*Gehyra mutilata*(UNCONFIRMED)
–	Body and tail covered with tubercles; coloration grey to brownish; very common in urban areas and plantations, rare in natural habitats	*Hemidactylus*, 34
34	Maximum snout-vent length of adults 55 mm; 2 to 8 rows of tubercles along the back, with 7 to 24 tubercles per row; dorsal side of body mostly uniformly white, beige or yellowish at night, pattern of dark stripes and spots visible during the day	*Hemidactylus frenatus* (AN, GC, MA, MO)
–	Pattern of dark stripes and spots mostly also visible at night; 8 or more rows of tubercles along the back, with 21 or more tubercles per row	35
35	Maximum snout-vent length of adults 51 mm; dorsal side covered with relatively prominent brown and whitish tubercles; 3 to 5 dorsal rows of brown spots often visible; 14 to 17 rows of tubercles along the back, with 23 to 30 tubercles per row	*Hemidactylus parvimaculatus* (AN, MO)
–	Snout-vent length of adults up to more than 51 mm; dorsal side usually with pattern of brighter and darker colors, but no whitish tubercles present	36
36	Maximum snout-vent length of adults 59 mm; original tail with whorls of spiny, enlarged scales extending far into the distal half of the tail; males with 25 to 32 femoral pores; 8 to 16 rows of tubercles along the back, with 25 to 37 tubercles per row; cranial depression extending anteriorly from between the eyes less distinct than in 36 B)	*Hemidactylus mercatorius* (AN, GC, MA, MO)
–	Maximum snout-vent length 85 mm; original tail with whorls of spiny, enlarged scales largely restricted to the proximal part of the tail; males with 41 to 59 femoral pores; 8 to 14 rows of tubercles along the back, with 21 to 31 tubercles per row; cranial depression extending anteriorly from between the eyes more distinct than in 36	*Hemidactylus platycephalus* (AN, GC, MA, MO)
37	Small, slender geckos, maximum snout-vent length 40.8 mm; flanks darker than dorsum, with sharpest contrast in head region; never with crossbands on body	*Ebenavia inunguis* (AN, GC, MA, MO)
–	Medium-sized geckos with robust heads, maximum snout-vent length 66.9 mm; four bright crossbands on dorsum, best visible at night; coloration of flanks not clearly distinguished from that of dorsum	*Paroedura sanctijohannis* (AN, GC, MA, MO)
38	Neck with crest of spiny scales; scales on tail similar to those on body; males with contrasting black and orange color pattern	*Agama agama* (GC)
–	Crest of spiny scales on neck absent; tail with enlarged scales, spiny	*Oplurus cuvieri comorensis* (GC)

## Discussion

### Unconfirmed records and taxonomic comments

For all the species records given in Table 1 at least one voucher specimen for each island was either collected during our surveys or traced in natural history collections. The only exception is *Hemidactylus mercatorius* on Mohéli, of which we do not know of any voucher specimen. The following species of terrestrial reptiles were previously recorded in the Comoros, but were not confirmed during our study.

***Lycodon aulicus***

Specimens of the Wolf snake *Lycodon aulicus* were neither available for examination nor observed. The locality of one specimen, CAS 135119, collected by E.J. Morris and traced by a search in the HerpNET database, is given as “Moroni, hospital ground North of Moroni”. During our field surveys, we searched at the hospital ground and interviewed employees, but could not find any evidence for an extant population of this species. Another specimen, also traced by HerpNET (FMNH 205907), was collected by J. Visser in 1973 on Grand Comoro, but no further information about this specimen is available. The presence of this species possibly resulted from a single introduction event and no evidence of an extant population of *Lycodon aulicus* is known to exist. *Lycodon aulicus capucinus*, now often considered as distinct species *Lycodon capucinus*, is an invasive species on the Mascarene islands (Cheke and Hume 2008).

***Lycodryas gaimardii* and *Lycodryas maculatus***

No unambiguous records of these species on the Comoros exist. Historical records and their possible taxonomic implications will be discussed in an upcoming paper on *Lycodryas sanctijohannis* (Hawlitschek et al., unpublished data, see also Hawlitschek 2008).

***Gehyra mutilata***

This nocturnal gecko, similar in appearance to the genus *Hemidactylus*, is widespread in the Indian Ocean area and considered an invasive species in many regions (Denny et al. 2005, Witmer et al. 2007, Rocha et al. 2009b). Its existence on the Comoros was apparently first suspected by Blanc (1971), without indicating any specific island, and subsequently by Cole (1992) and Paris (1999), but was never confirmed. During our field surveys in the Comoros, we did not detect any signs of the presence of this species, and did not find any voucher specimens in the studied collections.

***Meroles knoxii***

As described in Günther (1879), the single Anjouan specimen of the lacertid lizard *Meroles knoxii* was collected by C.E. Bewsher together with other material from Anjouan, including the type specimens of *Paroedura sanctijohannis* and *Lycodryas sanctijohannis*. While many localities from this period are doubtful, this latter fact supports the assumption that this specimen was indeed collected on Anjouan. However, as no other report of *Meroles knoxii* from the Comoros has come to be known since, the status of this species on Anjouan remains uncertain and its possible presence on the Comoros is in need of confirmation. For biogeographical reasons it is very unlikely that this species colonized the Comoros by natural dispersal but it may be that at some point, *Meroles knoxii* was introduced to this island but later became extinct. In another case, the scincid lizard *Trachylepis striata* remained hidden for several decades (e.g. Brygoo 1981, Meirte 2004), and it was rediscovered only in 2003 at Anjouan (Carretero et al. 2005). This demonstrates that the future rediscovery of species, whose occurrence on the Comoros is considered dubious is possible, and even the presence of yet unknown reptile species cannot be excluded.

***Hemidactylus mabouia* and *Hemidactylus brooki***

The Comorian populations of *Hemidactylus* which were formerly assigned to *Hemidactylus mabouia* (Blanc 1971, 1972, Meirte 2004) are now assigned to *Hemidactylus mercatorius* and *Hemidactylus platycephalus* (Vences et al. 2004, Carretero et al. 2005, Rocha et al. 2010b). The Comorian populations formerly assigned to *Hemidactylus brooki* (Carretero et al. 2005) are now assigned to *Hemidactylus parvimaculatus* (Bauer et al. 2010, Rösler and Glaw 2010).

### Land cover changes and state of the natural vegetation

Similar to other tropical oceanic islands, the Comoros were likely covered by large areas of forest before the arrival of man (Pascal 2002, Louette et al. 2004). Today, as shown by the present results, land cover of natural or near natural forest on the Comoros is classified at less than 7%. As the remote sensing classification relies mainly on photosynthetic activity and canopy closure, minor modifications from selective logging or agro-forestry may be undetected. This means that the less than 7% coverage of this class of forest contains pristine undisturbed forest but may also comprise areas where significant human impact is present.

Opening of the forest canopy is one of the prime factors inducing changes in forest microclimate and understory vegetation and thus affecting the species composition of the fauna (Chen et al. 1999, Jennings et al. 1999). Large areas still covered with forest vegetation are heavily degraded and their canopy has been opened. Areas where intensive logging has taken place are often cultivated with arborescent crops, small-scale plantations of other crops and neophyte stands. In lowland areas, forests have been replaced by extensive crop plantations. On Anjouan, Mayotte and Mohéli, the largest forest areas remain in the highest altitude ranges. Only on Grand Comoro, they are replaced by montane dry vegetation in altitudes too high for tropical forest (Figure 3).

Nearly a third of the area of the Comoros is covered by dry and low vegetation. This definition includes natural types of non-forest vegetation as well as crop stands and areas in which erosion and devegetation has led to soil fatigue to such an extent that crops cannot be grown (called “Padzas”). It can be assumed that even before the arrival of man, dry and low vegetation already made up a certain proportion of the Comorian vegetation. However, large areas which are now covered by dry / low vegetation were originally covered by dry forests, of which today only fragments are left (Pascal 2002, Louette et al. 2004).

### Habitat use and the impact of altitude

As mentioned above, it is assumed that the Comoros were originally covered largely by rainforest. This might lead to the assumption that forest and other original vegetation classes host a larger diversity than secondary vegetation, since plantation and degraded forest are the results of human impact of the last millennium. However, relatively low numbers of species were recorded in closed forest. This may be attributed to the relatively small area occupied by this habitat, but this alone cannot easily explain why fewer species were recorded in natural forests than in plantations, degraded forests, dry and low vegetation and even in urban areas.

Several reasons for this pattern can be discussed. (1) Introduced species, mainly among the genera *Hemidactylus* and *Phelsuma*, are adapted to anthropogenic habitats, abundant, and easy to observe, and therefore make up a large part of the total records. (2) As several studies have shown (Fredericksen and Fredericksen 2002, Bell and Donnelly 2006), degraded habitats generally seem to show a higher diversity especially of reptiles than undisturbed forest in similar areas. This is explained with the higher diversity in microhabitats, as patches of original forest remain very close to a system of clearings and secondary vegetation, and the higher proportion of insolated sites, which is important for ectothermic animals. (3) For similar reasons, many reptile species are less abundant in closed forest areas and prefer perch sites higher above the ground, where insolation is higher. This makes observation more difficult. (4) The possibility of extinction should be considered. Lowland forest areas have probably been deforested over a long period of time, and species restricted to these habitats may have already gone extinct. (5) At least on Grand Comoro, regular volcanic eruptions of the Mt. Karthala might have disturbed the evolution of reptile communities specialized on undisturbed forest.

**Figure 5.**
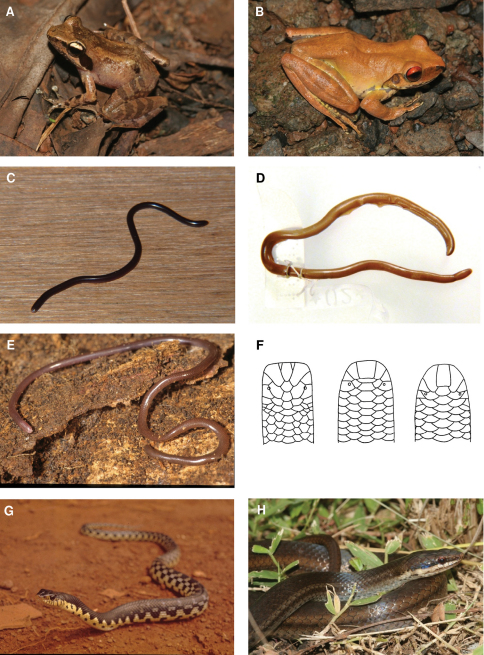
Species photographs. **A** *Blommersia* sp., ZSM 1706/2008, Mayotte **B** *Boophis* sp., ZSM 1711/2008, Mayotte **C** *Ramphotyphlops braminus*, ZSM 163/2010, Anjouan **D** *Typhlops comorensis*, MNHN 1895/126, Grand Comoro **E** *Typhlops* sp., ZSM 361/2002, Grand Comoro **F** drawings of heads of *Ramphotyphlops braminus*, *Typhlops comorensis* and *Typhlops* sp. (left to right) **G** *Leioheterodon madagascariensis*, Nosy Boraha, Madagascar **H** *Liophidium mayottensis*, ZSM 1693/2008, Mayotte.

Of the endemic species recorded, most are more abundant in secondary habitats than in pristine forest (Figure 5). The endemic reptiles *Amphiglossus johannae*, *Furcifer cephalolepis*, *Lycodryas sanctijohannis*, and *Phelsuma v-nigra* were all most commonly recorded in plantations or degraded forests. This is also true for both endemic amphibian species in Mayotte. There are only two endemic reptile species whose preference for pristine forest habitats can be supported by a significantly large number of records. These are the geckos *Paroedura sanctijohannis* and *Phelsuma nigristriata*. Nearly all *Phelsuma sanctijohannis* on Anjouan, Grand Comoro and Mohéli were observed in undisturbed forests, while on Mayotte this species was also commonly found in degraded habitats. *Phelsuma nigristriata* is known almost exclusively from pristine forests on Mayotte and largely depends on *Pandanus* plants as microhabitat which are restricted to relatively undisturbed forest. Only few, mainly juvenile, specimens were observed in neighbouring degraded habitats.

As shown in Figure 3, the majority of all species are most abundant in the low elevation ranges. However, a few endemic species are more common in or even confined to higher altitudes. Namely, these are *Phelsuma comorensis* and *Furcifer cephalolepis* on Grand Comoro and *Phelsuma nigristriata* and *Phelsuma pasteuri* on Mayotte. *Paroedura sanctijohannis* is almost exclusively found at high altitudes on all islands but Mayotte, where it covers the entire altitudinal range. In *Paroedura sanctijohannis* and *Phelsuma nigristriata*,this preference for higher altitudes can probably be explained by the availability of suitable habitat, since as stated above these are species more or less dependent on forest. Climate may also play a role especially for *Phelsuma nigristriata*, *Phelsuma comorensis* and *Phelsuma pasteuri* (B. Brückmann et al., unpublished data).

### Potential factors of threat for endemic Comorian reptiles and amphibians

The majority of endemics among the Comorian herpetofauna appear to have adapted to the habitat changes imposed by man. *Paroedura sanctijohannis* and *Phelsuma nigristriata* are the only species for which a true dependency on pristine forests can be postulated. Even these two species can also be found in degraded habitats, albeit in lower densities.

A much more potent threat for Comorian endemics may be competition by invasive species. In 1998, according to Meirte (2004), the agamid lizard *Agama agama* was introduced to Grand Comoro. It has since established a dense population in the capital Moroni, but has not yet been observed outside urban areas. While it may be a competitor to or even predator on other local species, the species most affected may be the Comoro iguana *Oplurus cuvieri comorensis*, which resembles the agama in habitus and ecology. The Comoro iguana inhabits only a narrow strip of rocky cliffs along the northeast coast of Grand Comoro. If the agama expands its range and reaches that of the Comoro iguana, there would be no areas of refuge for the latter species. If both species prove to be direct competitors, this might lead to the extinction of *Oplurus cuvieri comorensis*.

An even more evident situation of direct competition is that of the diurnal *Phelsuma* geckos, which are present on the Comoros with five endemic and two introduced species. While *Phelsuma comorensis* on Grand Comoro, as well as the Mayottean species, seem to prefer habitats where invasive *Phelsuma* species are rare or absent, *Phelsuma v-nigra* resembles the invasive species in terms of distribution and preferences. Focused studies are necessary to address the situation of competition between the various Comorian *Phelsuma* species.

The overwhelming majority of nocturnal geckos that can be observed on the Comoros belong to introduced species. These are the four species of *Hemidactylus*, all of which cover more or less extensive distribution ranges in the tropics. Three of these species are rarely seen outside urban and plantation areas of low altitudes, but *Hemidactylus platycephalus* has colonized all available types of habitats including undisturbed forests and has been observed in altitudes of over 600 m. On Anjouan, Grand Comoro and Mohéli, the endemic and nocturnal *Paroedura sanctijohannis* inhabits almost exclusively areas above this altitude. This could be explained by possible exclusion from lower altitudes through competition from *Hemidactylus platycephalus*. Only on Mayotte, *Paroedura sanctijohannis* regularly occurs in syntopy with *Hemidactylus platycephalus*.

While *Paroedura sanctijohannis* currently inhabits areas in elevations too high for competing invasive species, this may change if global warming would allow these competitors to colonize areas at higher altitudes. Raxworthy et al. (2008) describe the phenomenon of “upslope displacement” of habitat areas, which particularly can affect endemic highland species often restricted to mountaintops. With an increase in temperature, the lower distribution limit of these species rises; if it rises up to a level higher than the point of highest elevation in the species’ habitat area, the entire habitat of the concerned species is lost, leading to extinction.

No studies have yet been undertaken concerning the impact of predatory mammals on the Comorian herpetofauna. Tenrecs (*Tenrec ecaudatus*) inhabit all islands of the Comoro archipelago and were probably introduced in historical times (Louette et al. 2004), although this is unclear. This species mainly feeds on invertebrates but to some extent also on small, ground-dwelling vertebrates. On the Comoros, typhlopid snakes, skinks and frogs might form part of its nurriture. Two introduced species of viverroid carnivores may be stronger predators on reptiles: the Small Indian Civet (*Viverricula indica*) on Mayotte and the abundant Small Asian Mongoose (*Herpestes javanicus auropunctatus*) on Grand Comoro. They prey on a variety of small animals; in both species, insects and other invertebrates make up the largest part of the diet, but they also feed on amphibians and reptiles, including snakes (see Chuang and Lee 1997 for *Viverricula* and summary in Hays and Conant 2007 for *Herpestes*). Thus, both species, although this has not yet been proven, may also prey on reptiles, especially snakes, on the Comoros and therefore reduce the populations of these reptile species. The species most strongly affected might be the terrestrial Mayotte endemic snake *Liophidium mayottensis*.

These observations suggest that competition and predation by invasive species pose a more serious threat to Comorian endemic reptiles than destruction of the remaining natural habitats. However, habitat degradation and destruction, extension of settlements and intensification of land use also create more suitable conditions for invasive species and should therefore also be considered an important threat factor.

Several endemic Comorian reptile species have been subject to international trade as pets. Data are available only for species listed on the CITES appendices (UNEP-WCMC 2011). According to this database, a considerable number of animals was exported from the three islands of the Union of the Comoros. 22004 specimens of the day gecko *Phelsuma comorensis* and 23850 specimens of *Paroedura v-nigra* are listed for the period of 2000 to 2010. The numbers for Mayotte endemics are much lower at 2000 specimens for *Paroedura nigristriata*, 100 for *Paroedura pasteuri* and 280 for *Paroedura robertmertensi*. The situation of the chameleons of the genus *Furcifer* is similar: 14420 specimens of *Furcifer cephalolepis* from Grand Comoro were listed as exported since 2000, but only 1560 specimens of *Furcifer polleni* from Mayotte.

The relatively low export numbers of species from Mayotte are unlikely to have a significant impact on populations. For species endemic to the islands of the Union of the Comoros, these numbers are much higher. In the Union of the Comoros, little effort is required to obtain export permits for CITES-listed species in relation to Madagascar or Mayotte, where recent quotas for commercial export numbers are relatively low or even zero for most species. This condition may have directed pet traders to exploit populations in the Union of the Comoros more intensively. For comparison, 16252 *Phelsuma madagascariensis* and 14963 *Paroedura laticauda*, among the most popular vivarium species of the genus *Phelsuma*, are listed in the same period (2000 to 2010). These numbers are lower than those from the Comorian species. Notably, in the period of 1990 to 1999, export numbers of the Malagasy species were five-fold higher than in the following decade (91083 and 95391, respectively). The decrease of export in Malagasy species may have been a further incentive to exploit the populations of other countries.

It is difficult to assess the impact of the export of specimens for pet trade on the wild populations of the species concerned. Our results show that the relatively heavily exploited species *Phelsuma comorensis* and *Paroedura v-nigra* are more abundant in their natural habitats than the less heavily exploited species from Mayotte. This suggests that the collection of specimens so far did not have a significantly negative effect on their populations. The chameleon *Furcifer cephalolepis* from Grand Comoro, which has been much more intensely exploited than its relative from Mayotte, *Furcifer polleni*, is also less often observed in the wild than its congener. This could indicate that collection of specimens for pet trade in high numbers may negatively influence the populations of *Furcifer cephalolepis*. Since no reliable data on the abundance of this species before its exploitation is available, we have no means of investigating this speculation.

Amphibians are globally threatened by the chytrid fungus *Batrachochytrium dendrobatidis* (Daszak et al. 2003, Weldon et al. 2004, Mendelson et al. 2006, Skerratt et al. 2007, Wake and Vredenburg 2008, Rödder et al. 2009), which has led to massive reductions and even extinctions of amphibian populations. So far, no amphibian population declines have been noticed on Mayotte and surveys for this fungus have not been conducted. It is unknown how strongly it might affect the endemic anuran species, but reviewing its impact on other amphibian species worldwide, it must be assumed to cause severe losses among Mayotte’s amphibians if introduced.

### Conservation assessments

We evaluate all species of reptiles and amphibians endemic to the Comoros against the criteria for Red List categories in IUCN (2001a). These assessments are proposals for national conservation status and are also currently under review at the CI-IUCN Biodiversity Assessment Unit for inclusion in the IUCN Red List (P. Bowles, pers. comm. 2011-05-17 to 2011-09-06). Some endemic reptile species from the Comoros were previously evaluated by other authors and were all given the status “Least Concern”. Our results do not always confirm these assessments, and we propose changing the Red List entries to better reflect the threat situations of these species.

We also present an assessment of *Trachylepis comorensis* although a population of this species is known on the islet of Nosy Tanikely, Madagascar (Köhler et al. 1997). Additionally, genetic studies by Mausfeld-Lafdhiya et al. (2004) and Rocha et al. (2010a) show that the subspecies *Trachylepis maculilabris casuarinae* from Casuarina Island off the coast of North Mozambique is nested within *Trachylepis comorensis*. According to these studies, however, the two non-Comorian areas were probably colonized with human aid, and the Comoros are the centre as well as the original distribution range of *Trachylepis comorensis*.

Taxonomic units below species level currently do not receive any status in the IUCN Red List. Nevertheless, we present assessments for the endemic subspecies of *Oplurus cuvieri* and *Cryptoblepharus boutonii*. The Comoro Iguana *Oplurus cuvieri comorensis* was already considered a separate species by (Meirte (1992, 2004). Münchenberg et al. (2008) later found no genetic divergence between the Comorian and the Malagasy *Oplurus cuvieri* and it is now considered a subspecies. We agree with this view but still evaluate the Red List status of *Oplurus cuvieri comorensis* because we recognize the differences in morphology and behaviour to the nominate form (Meirte 1992) and because, according to our evaluation, it is the most threatened reptile taxon on the Comoros. Several subspecies of *Cryptoblepharus boutonii*, including those from the Comoros, have been treated as species (Horner 2007). We follow Rocha et al. (2006) in considering all WIO forms as subspecies.

Although IUCN does not support Red List assessments for undescribed species, we here provide an evaluation of their national conservation status applying the Red List Criteria, in order to help inform assessment of these species once they are formally described.

A summary of the assessments is given in Table 3. Future taxonomic studies may reveal further endemic species on the Comoros, possibly in the genera *Ebenavia*, *Geckolepis*, *Lycodryas* and *Paroedura*.

**Table 3.** Proposed Red List status, extent of occurrence (EOO) and area of occupancy (AOO) of reptile and amphibian species endemic to the Comoros. EOO and AOO are given in sqkm. Endemic subspecies that are not endemic at species level are marked with an asterisk (*).

*Species*	*Status*	*EOO*	*AOO*
*Typhlops comorensis*	DD	1433.5	?
*Typhlops* sp.	DD	1009.9	?
*Liophidium mayottensis*	EN B1abiii + B2abiii	376.0	65.2
*Lycodryas sanctijohannis*	NT	2025.0	1737.4
*Furcifer cephalolepis*	LC	1009.9	718.1
*Furcifer polleni*	LC	376.0	371.5
*Paroedura sanctijohannis*	EN B1abiii + B2abiii	2025.0	60.8
*Phelsuma comorensis*	NT	250.1	183.5
*Phelsuma nigristriata*	VU D2	376.0	24.8
*Phelsuma pasteuri*	NT	376.0	127.0
*Phelsuma robertmertensi*	EN B1abiii + B2abiii	376.0	98.9
*Phelsuma v-nigra*	NT	1650.1	1368.2
*Oplurus cuvieri comorensis* *	CR B1abv + B2abv	7.6	<7.6
*Amphiglossus johannae*	LC	2025.0	1737.4
*Cryptoblepharus boutonii ater* *	LC	?	?
*Cryptoblepharus boutonii degrijsii* *	LC	?	?
*Cryptoblepharus boutonii mayottensis* *	NT	?	?
*Cryptoblepharus boutonii mohelicus* *	LC	?	?
*Trachylepis comorensis*	LC	>2025.0	>1811.0
*Blommersia* sp.	NT	376.0	297.6
*Boophis* sp.	NT	376.0	297.6

***Oplurus cuvieri comorensis***

This iguana inhabits only rocky shores in a very small area on Grand Comoro. We measured the length of coastline potentially inhabitable for *Oplurus cuvieri comorensis* as 38 km. Assuming that the iguana inhabits areas as far away as 200 m from the coast, which is probably not true for most of its range, this would mean an extent of occurrence of roughly 7.6 sqkm. The actually inhabited area (area of occupancy) is even smaller, as not all coastal areas are colonized. The two foremost threatening factors are competition with invasive species and collection of individuals by locals for food and entertainment (Meirte 2004 and pers. obs.), which cause a continuing decline in number of mature individuals (CR B1bv + B2bv). If the invasive *Agama agama* extends its range to the North of Grand Comoro, the entire range of *Oplurus cuvieri comorensis* will be affected.

According to IUCN (2001a), “locations” are areas where “a single threatening event can rapidly affect all individuals of the taxon present”. Thus, all known localities of *Oplurus cuvieri comorensis* can be considered a single location (CR B1a+B2a) because of the uniformity of the habitat and the short distances in between. According to the facts presented, the status of Critically Endangered (CR B1abv + B2abv) is proposed for *Oplurus cuvieri comorensis*.

***Typhlops comorensis* and *Typhlops* sp.**

Due to their cryptic and fossorial habits virtually nothing is known on the biology, distribution and ecology of both putatively endemic *Typhlops* species. *Typhlops comorensis* is known from only a few specimens, and only two were recorded in our surveys. *Typhlops* sp. is a very tiny species known from only very few individuals (including ZSM 361/2002 and 362/2002) from the area around Moroni, the capital of Grand Comoro. It is likely to represent a new, undescribed species (Wallach pers. comm.) but current knowledge does not exclude the possibility that this species has been accidentally introduced from somewhere else. There are no indications that these two species are restricted to the remaining undisturbed forest areas or that *Ramphotyphlops braminus* or any other possibly introduced species impose high pressure on them. With regards to the poor knowledge available the status Data Deficient appears appropriate for both species.

***Liophidium mayottensis***

Only two localities of this terrestrial and diurnal snake endemic to Mayotte were available for this analysis and therefore no specific statements about its biology, distribution and population size can be made. As no specific data on the distribution of this snake is available and one of the two specimens was caught in forest, it should be assumed that *Liophidium mayottensis* will be affected by a further degradation and fragmentation of its habitat and probably also by the introduced carnivorous Small Indian Civet (*Viverricula indica*). While the total area of Mayotte (376.0 sqkm) is recognized as extent of occurrence, we conservatively assume that *Liophidium mayottensis* is restricted to an area in central Mayotte forming a single location, where both observations were made. Assuming that plantation and closed forest, where specimens have been observed, and degraded forest as “intermediate” are suitable habitat classes, we calculate an area of occupancy of 65.2 sqkm. Therefore, the status Endangered (EN B1abiii + B2abiii) is applied for this very rarely encountered and poorly known species that should become a focal species for future studies and monitoring programs.

***Lycodryas sanctijohannis***

This largely arboreal and nocturnal snake was observed in a variety of habitat classes on all islands (extent of occurrence 2025.0 sqkm, area of occupancy 1737.4 sqkm) and seems to be well adapted to living in natural forests as well as in plantation and urban areas. Its French vernacular name “serpent des cocotiers” (coconut palm snake) was probably given by locals who frequently encounter this snake while collecting coconuts. Thus, further destruction of the remaining natural vegetation is unlikely to affect the species’ area of occurrence. Considering the crepuscular and mostly tree dwelling habits of this snake, the low number of observations does not necessarily point to a population decline or general rareness. The fact that locals state to observe, on average, about one snake per day per village (pers. observation), may even mean that *Liophidium sanctijohannis* is still relatively abundant. Potential threats for this species may come in further extension of the infrastructure in settlements and intensification of horticulture, which may lead to the result that these areas are less suitable as habitats for *Liophidium sanctijohannis*, and subpopulations will be isolated from each other by habitat fragmentation(ENB1biii). Snakes are regularly killed by farmers and planters, and extension of settlements and intensification of horticulture will also increase this threat factor. Since none of these threats is imminent and the species is unlikely to be strongly affected by the introduced species (carnivores, rats, tenrecs, other reptiles or birds) at present, the status Near Threatened is proposed.

***Furcifer cephalolepis***

Current listing:Least Concern (Carpenter 2009)

This chameleon inhabits various habitats on Grand Comoro, in an area of occupancy of 718.1 sqkm. It is most common at the mountain of La Grille. Since *Furcifer cephalolepis* is also abundant in plantation and degraded forest, deforestation cannot be considered an imminent threat. However, relatively high export numbers from 2000 to 2010 could have had a negative impact on this species, although this cannot be proven at present. We therefore propose to maintain the status Least Concern, but note that future monitoring programs, if implemented, should also regard this species.

***Furcifer polleni***

Although limited to a much smaller extent of occurrence and thus also area of occupancy (371.5 sqkm) than *Furcifer cephalolepis*, *Furcifer polleni* from Mayotte island is more commonly observed and inhabits a variety of habitats from pristine forest to gardens in densely populated areas. (Blanc (1971, 1972, and cited in Raxworthy et al. 2002) reports *Furcifer polleni* for Mohéli, but not for Mayotte. We have found no indications of a presence of this species on Mohéli and consider this record erroneous, in accordance with Meirte (2004). An allochthonous population on Anjouan, which is not considered for the calculation of area of occurrency, is so far only known from gardens in the town of Hombo (Meirte 2004 and pers. obs.). Export numbers from 2000 to 2010 were relatively low (1562 specimens) and significant threats were not observed. We propose to assign the status Least Concern to *Furcifer polleni*.

***Paroedura sanctijohannis***

This nocturnal gecko is exposed to a greater risk than many other Comorian reptile species. While the Mayotte population shows no clear preference for any habitat or altitudinal range, the populations of the other three islands are almost exclusively restricted to high altitude forest which is subject to ongoing deforestation. Another potential threat to which *Paroedura sanctijohannis* may be subjected in the future, due to its apparent restriction to mountain summits, is upslope displacement of its suitable habitats induced by global warming (Raxworthy et al. 2008). Increasing temperatures may also lead to an upslope displacement of its potential invasive competitor *Hemidactylus platycephalus* which is currently restricted to elevations below the distribution range of *Paroedura sanctijohannis* on Grand Comoro, Mohéli, and Anjouan. As the area recorded for *Paroedura sanctijohannis* on these three islands is already reduced to a narrow range just below the summits of the mountains concerned, there is little buffer zone for displacement left. While the extent of occurrence is calculated to be 2025.0 sqkm, the area of occupancy is much smaller. Since mostly erratic juvenile specimens were observed outside closed forest, we assume that this habitat is not suitable for maintaining viable and reproducing populations. We calculate the area of occupancy as the sum of all areas of closed forest in which *Paroedura sanctijohannis* was observed, plus the dry vegetation on Saziley peninsula of Mayotte, resulting in a severly fragmented area of 60.8 sqkm. This qualifies for the category Endangered, although it is much closer to Critically Endangered (below 10 sqkm) than to Vulnerable (500 sqkm). The status Endangered (EN B1abiii + B2abiii) is proposed for *Paroedura sanctijohannis*.

***Phelsuma comorensis***

Current listing:Least Concern (Ineich 2009b)

This day gecko is restricted to a relatively small area on Grand Comoro, the mountain of La Grille, where it can be found in considerable abundance in degraded forests as well as in areas which are subject to heavy human influence, including banana plantations and open urban areas. An erratic individual was also observed near the capital Moroni, but no populations outside the La Grille area are known. Therefore, an extent of occurrence of 250.1 sqkm and an area of occupancy of 183.5 sqkm are calculated (EN B1biii+B2biii), and La Grille can be considered a single location (EN B1a+B2a). *Phelsuma comorensis* lives syntopically with the invasive species *Phelsuma dubia* throughout most of its range, although strong competition has not been observed so far. Further human development and population growth may lead to intensification of agriculture and horticulture, and to unpredictable general changes in land use including intensified use of pesticides which could lead to strong degradation of its area of occupancy. This would qualify the species for the status Endangered (EN B1abiii + B2abiii). We therefore propose the status Near Threatened.

***Phelsuma nigristriata***

As discussed above, among the Comorian day geckos *Phelsuma nigristriata* is the species most dependant on the remaining natural forest vegetation. Excluding the altitude level below 100 meters, where *Phelsuma nigristriata* was not observed, the remaining area covered by the habitat class forest on Mayotte is calculated as 24.83 sqkm. This qualifies for the IUCN criteria Endangered with less than 500 sqkm (EN B2), but is relatively close to the limit of 10 sqkm for the status Critically Endangered (CR B2). A small number of presumably erratic juvenile specimens was also observed in degraded forest, however, we assume that natural forests are necessary to maintain viable and reproducing populations. The forest areas are severly fragmented, and due to the high rates of human population growth on Mayotte, there is a high risk that increased degradation of pristine forests will lead to a continuing decline in the extent and quality of this habitat. This might drive the population of *Phelsuma nigristriata* to Critically Endangered in a very short time (criterion VU D2). We therefore propose the status Vulnerable (VU D2) for this species.

***Phelsuma pasteuri***

Among the species of *Phelsuma* endemic to Mayotte, *Phelsuma pasteuri* appears to be best adapted to degraded habitats, as discussed above. In areas of human influence, especially plantations, there is potential high competitive pressure imposed by *Phelsuma laticauda*, thus natural forest should be seen as the more adequate habitat for *Phelsuma pasteuri*. We calculate an area of occupancy of 127.0 sqkm, which brings this species close to meeting the criteria for the status Endangered (EN B1abiii + B2abiii). Only few specimens were exported for the pet trade (100 specimens). Currently, the status Near Threatened should be applied.

***Phelsuma robertmertensi***

As discussed above, *Phelsuma robertmertensi* is found mainly in mangroves and coastal tree stands, but also in the forest at Choungui on Mayotte. Despite the formal protection of mangroves and forests on Mayotte, these preferred habitats should still be considered threatened by human activities and fragmentation. An area of occupancy of 98.9 sqkm is calculated for *Phelsuma robertmertensi*. Almost the entire population of this species inhabits areas where individuals are exposed to competition by the introduced *Phelsuma laticauda*. As defined by this threat, the species is considered to occur at a single location. The status Endangered (EN B1abiii + B2abiii) should be assigned.

***Phelsuma v-nigra***

Current listing:Least Concern (Ineich 2009c)

On all the islands where *Phelsuma v-nigra* subspecies occur, they inhabit a variety of habitats in a total area of occupancy of 1368.2 sqkm and live in sympatry with at least one introduced species of *Phelsuma*. Though partially more abundant in higher altitude zones than the competitor *Phelsuma dubia* (and probably also *Phelsuma laticauda* on Anjouan), as shown by the results discussed above, no evidence clearly points to a decline of this species (EN B1bv). Elucidating this situation by long-term observations would be desirable. Moreover, many localities of *Phelsuma v-nigra* are situated in plantations and urban areas, so currently the area suitable for this species does not appear to be subjected to fragmentation and degradation (EN B1abiii). Therefore, the status Near Threatened (EN B1abiii + B1bv) appears appropriate for this species and should also be applied to all subspecies.

***Amphiglossus johannae***

Current listing:Least Concern (Ineich and Meirte 2009)

*Amphiglossus johannae* was observed on all islands in many different habitat classes, including habitats under human influence, and altitudes. Given the cryptic and fossorial habits of this skink a relatively high abundance cannot be excluded. No threat factors are currently visible. The status Least Concern should be maintained.

***Cryptoblepharus boutonii* subspecies**

Current listing: *Cryptoblepharus ater*: Least Concern (Ineich 2009a)

*Cryptoblepharus boutonii* subspecies occupy an ecological niche unusual for reptiles. They live in rocky tidal zones, a habitat comprising little area and subjected to continuous change, as suitable areas may appear and disappear with erosion and tides. It is difficult to measure extent of occurrence and area of occupancy using our methodology, since rocky tidal zones are patchy and hard to detect using Landsat imagery. The subspecies *Cryptoblepharus boutonii ater* (Grand Comoro), *Cryptoblepharus boutonii degrijsii* (Anjouan) and *Cryptoblepharus boutonii mohelicus* (Mohéli) are regularly observed in suitable habitats, also inhabit harbours and shores near settlements and are common in syntopy with potentially competitive *Trachylepis comorensis* and – on Grand Comoro – *Agama agama*. We propose the status Least Concern for these subspecies. In contrast, *Cryptoblepharus boutonii mayottensis* in Mayotte could often not be detected in seemingly suitable habitats and was never recorded in harbours, which raises the suspicion of a past decline in area, extent or quality of its habitat (EN B1biii). We propose the status Near Threatened for this subspecies.

***Trachylepis comorensis***

On all islands, *Trachylepis comorensis* is widespread and occurs in primary forest as well as in areas under heavy human influence. In addition to the Comoros, it inhabits only small islets. No signs of reduction or fragmentation of the area of occupancy or population decline can be detected. The status Least Concern is applied to this species.

***Blommersia* sp.and** ***Boophis* sp.**

Both anuran species treated here were recorded from various habitat classes, mainly classes under heavy human influence. Areas of occupancy of 297.6 sqkm were calculated for both species. Threats by habitat degradation or population declines can neither be observed nor inferred. However, the populations of both species might rapidly break down in the case of the introduction of the amphibian chytrid fungus, which is considered one of the prime factors of the global amphibian decline as has already been introduced to many previously unaffected areas (Daszak et al. 2003, Weldon et al. 2004, Mendelson et al. 2006, Skerratt et al. 2007, Wake and Vredenburg 2008, Rödder et al. 2009). This might lead to a classification of Endangered (EN B1bv + B2bv). At present, the status Near Threatened should be applied for both species.

**Figure 6.**
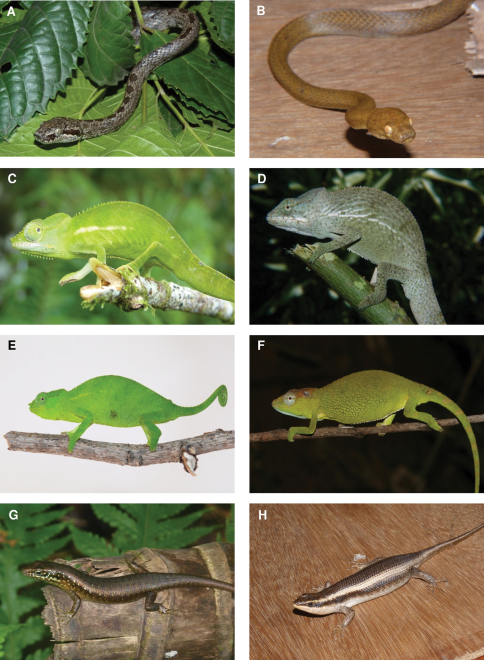
Species photographs. **A** *Lycodryas sanctijohannis*, male, ZSM 38/2010, Anjouan **B** *Lycodryas sanctijohannis*, female, ZSM 40/2010, Anjouan **C** *Furcifer cephalolepis*, male, Grand Comoro **D** *Furcifer polleni*, male, Anjouan **E** *Furcifer cephalolepis*, female, Grand Comoro **F** *Furcifer polleni*, female, Mayotte **G** *Trachylepis comorensis*, Mohéli **H** *Trachylepis striata*, ZSM 70/2010, Anjouan.

**Figure 7.**
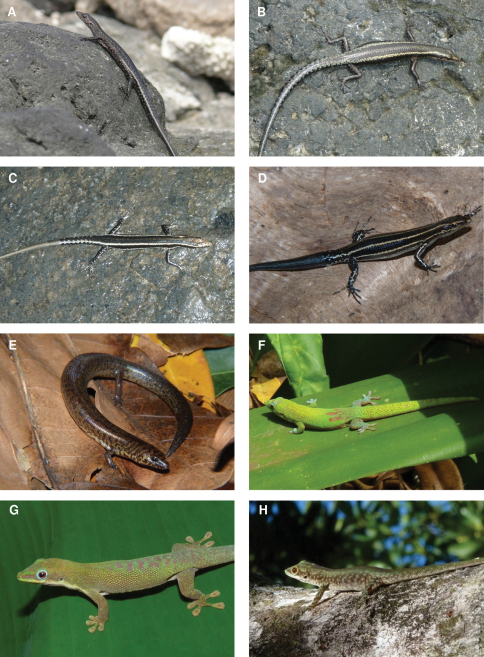
Species photographs. **A** *Cryptoblepharus boutonii ater*, Grand Comoro **B** *Cryptoblepharus boutonii mohelicus*, ZSM 1680/2008, Mohéli **C** *Cryptoblepharus boutonii mayottensis*, ZSM 1703/2008, Mayotte **D** *Cryptoblepharus boutonii degrijsii*, ZSM 63/2010, Anjouan **E** *Amphiglossus johannae*, ZSM 54/2010, Anjouan **F** *Phelsuma laticauda*, Anjouan **G** *Phelsuma dubia*, Mayotte **H** *Phelsuma robertmertensi*, Mayotte.

**Figure 8.**
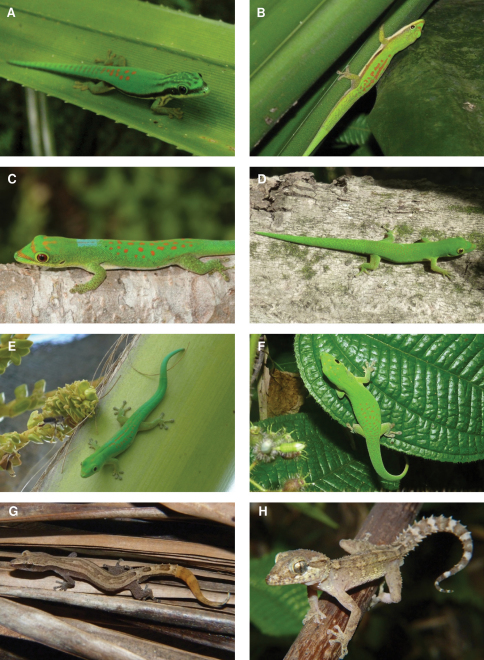
Species photographs. **A**. *Phelsuma nigristriata*, Mayotte **B** *Phelsuma comorensis*, Grand Comoro **C** *Phelsuma pasteuri*, Mayotte **D** *Phelsuma v-nigra anjouanensis*, Anjouan **E** *Phelsuma v-nigra comoraegrandensis*, Grand Comoro **F** *Phelsuma v-nigra v-nigra*, Mohéli **G** *Ebenavia inunguis*, ZSM 68/2010, Anjouan **H** *Paroedura sanctijohannis*, ZSM 98/2010, Mayotte.

**Figure 9.**
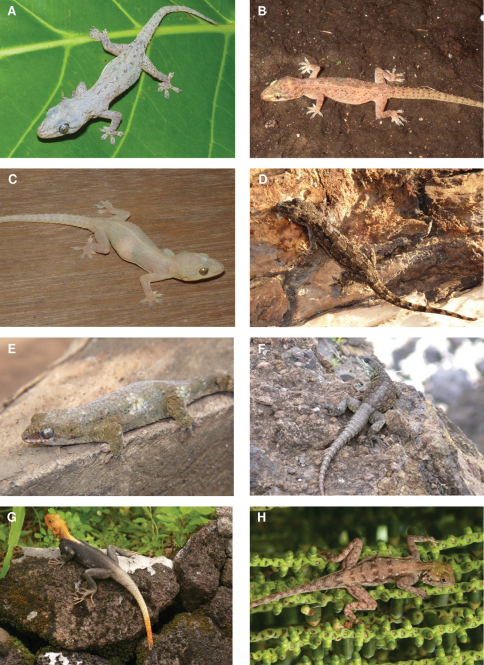
Species photographs. **A** *Hemidactylus frenatus*, ZSM 116/2010, Mohéli **B** *Hemidactylus parvimaculatus*, ZSM 370/2002, Mohéli **C** *Hemidactylus mercatorius*, ZSM 121/2010, Anjouan **D** *Hemidactylus platycephalus*, Anjouan **E** *Geckolepis maculata*, ZSM 83/2010, Anjouan **F** *Oplurus cuvieri comorensis*, Grand Comoro **G** *Agama agama*, male, Grand Comoro **H** *Agama agama*, juvenile, Grand Comoro.

**Figure 10.**
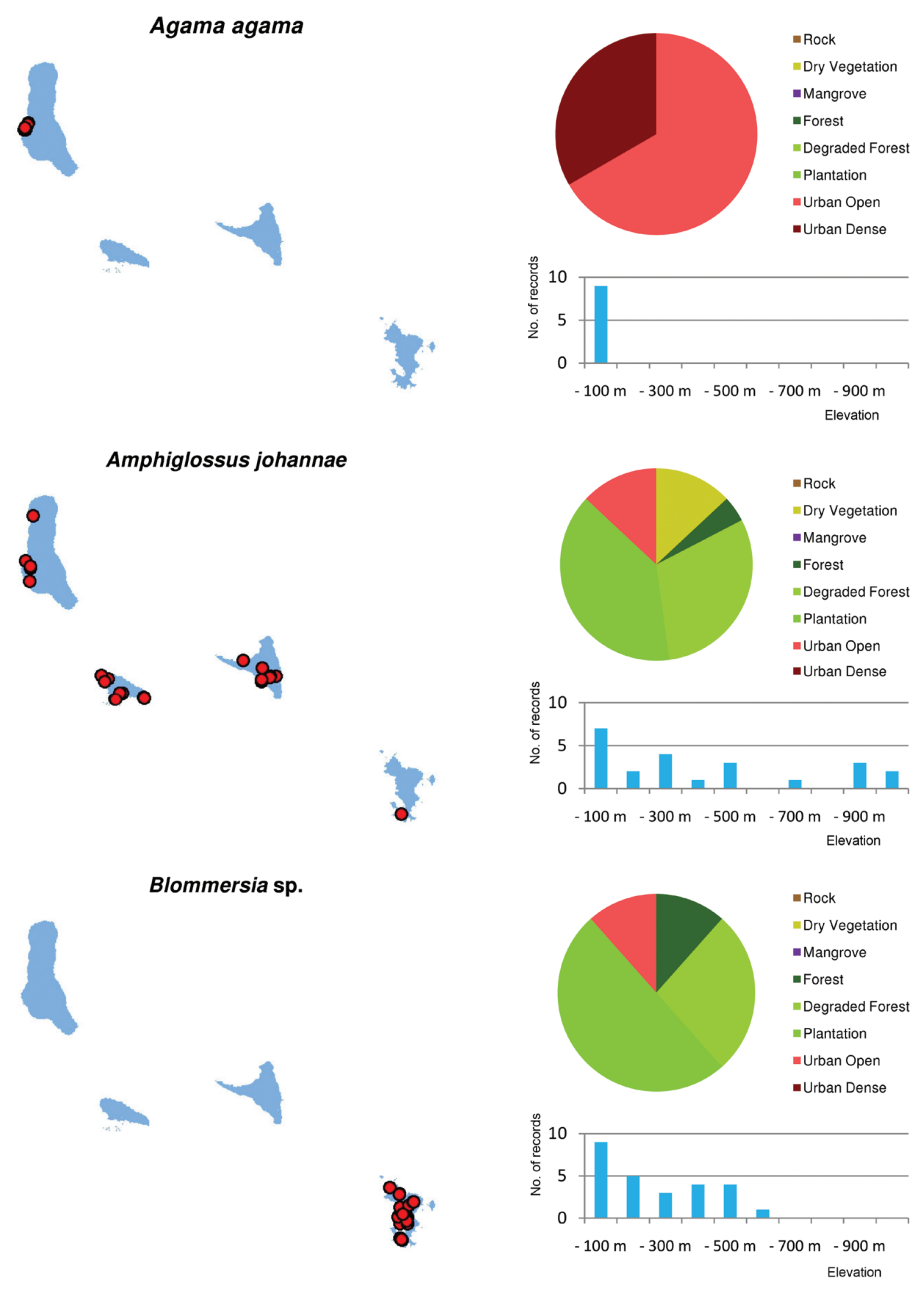
Distribution maps, and distribution over habitat and altitude classes, for *Agama agama*, *Amphiglossus johannae* and *Blommersia* sp.

**Figure 11.**
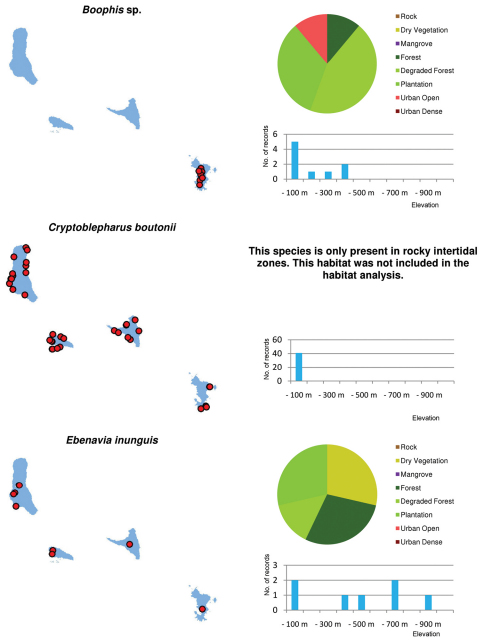
Distribution maps, and distribution over habitat and altitude classes, for *Boophis* sp., *Cryptoblepharus boutonii* and *Ebenavia inunguis*.

**Figure 12.**
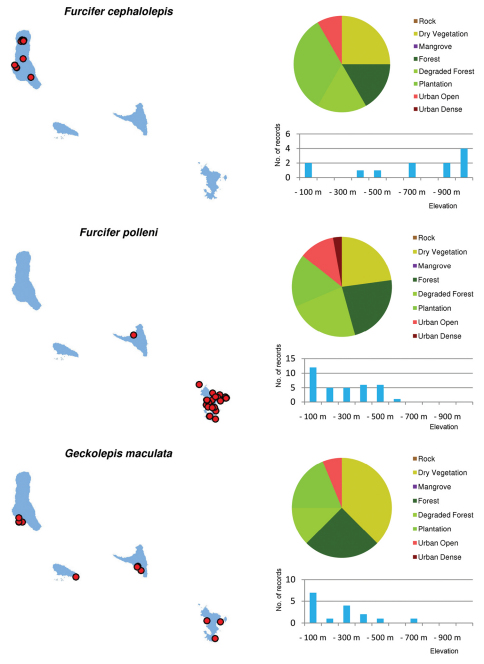
Distribution maps, and distribution over habitat and altitude classes, for *Furcifer cephalolepis*, *Furcifer polleni* and *Geckolepis maculata*.

**Figure 13.**
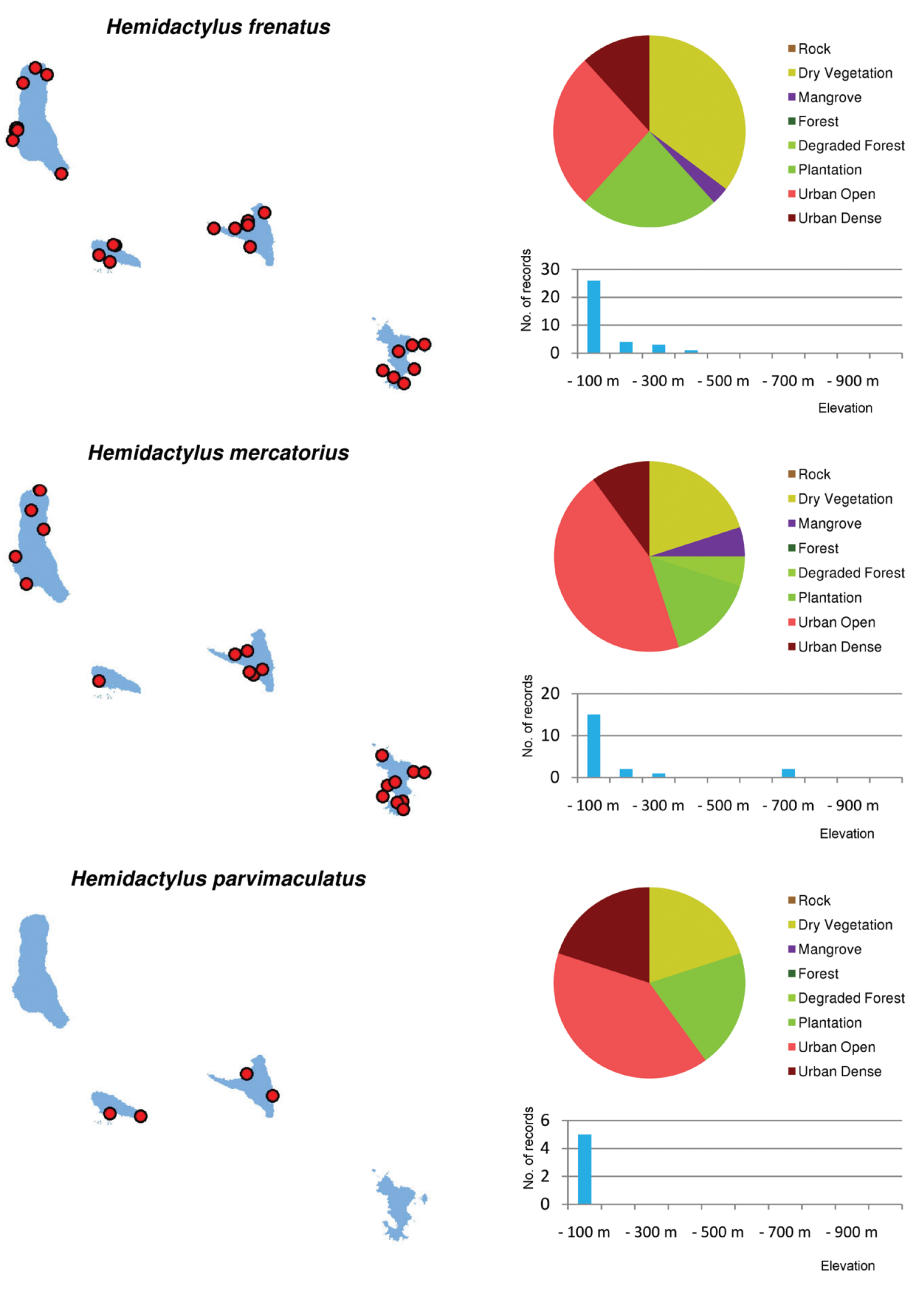
Distribution maps, and distribution over habitat and altitude classes, for *Hemidactylus frenatus*, *Hemidactylus mercatorius* and *Hemidactylus parvimaculatus*.

**Figure 14.**
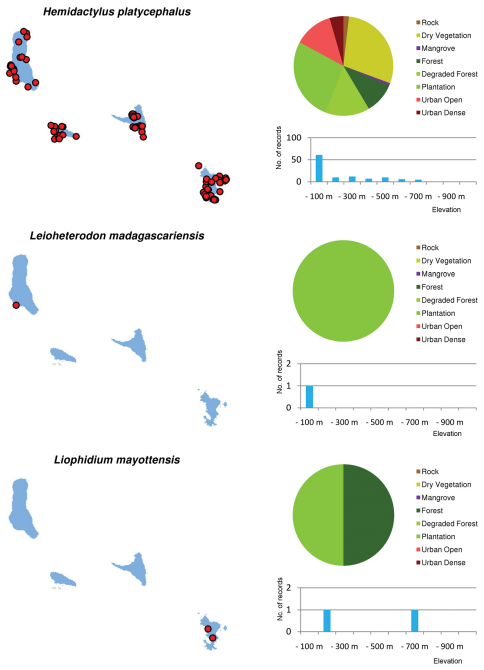
Distribution maps, and distribution over habitat and altitude classes, for *Hemidactylus platycephalus*, *Leioheterodon madagascariensis* and *Liophidium mayottensis*.

**Figure 15.**
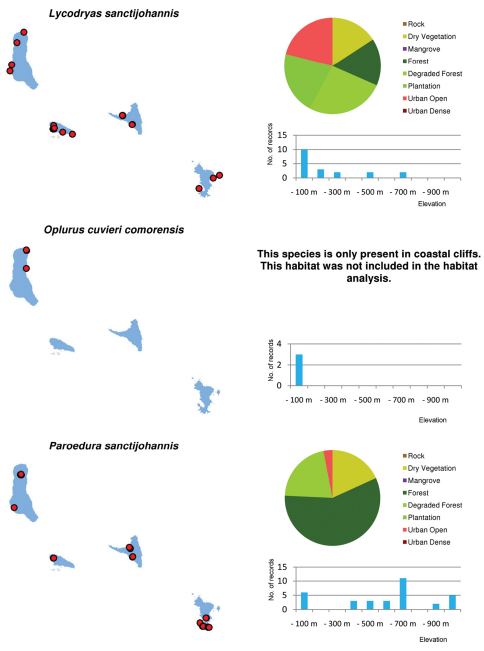
Distribution maps, and distribution over habitat and altitude classes, for *Lycodryas sanctijohannis*, *Oplurus cuvieri comorensis* and *Paroedura sanctijohannis*.

**Figure 16.**
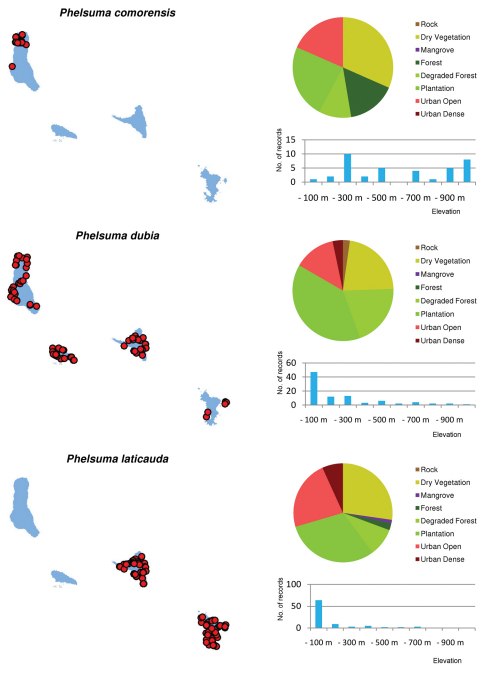
Distribution maps, and distribution over habitat and altitude classes, for *Phelsuma comorensis*, *Phelsuma dubia* and *Phelsuma laticauda*.

**Figure 17.**
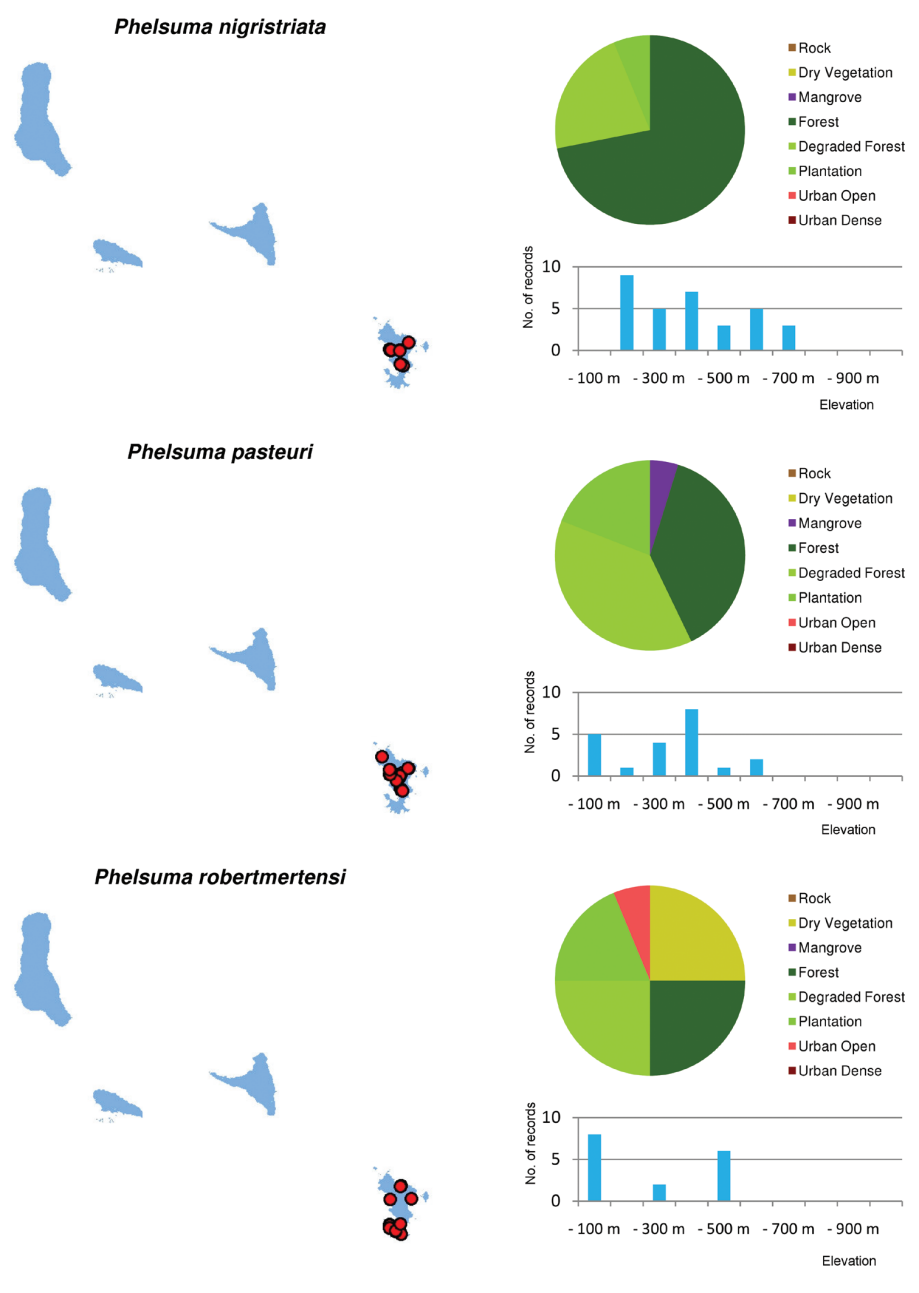
Distribution maps, and distribution over habitat and altitude classes, for *Phelsuma nigristriata*, *Phelsuma pasteuri* and *Phelsuma robertmertensi*.

**Figure 18.**
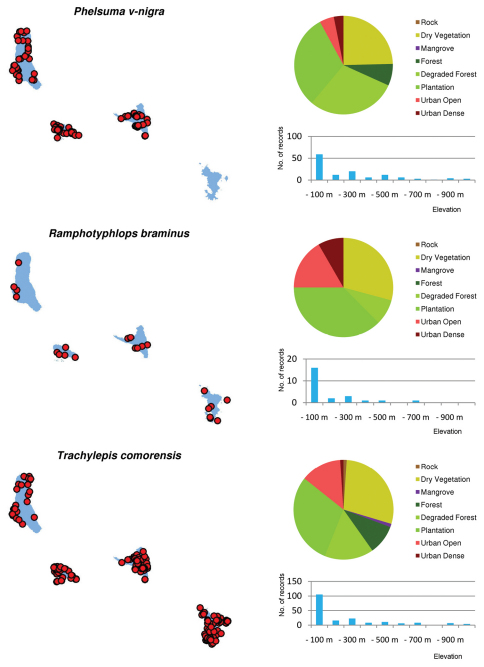
Distribution maps, and distribution over habitat and altitude classes, for *Phelsuma v-nigra*, *Ramphotyphlops braminus* and *Trachylepis comorensis*.

**Figure 19.**
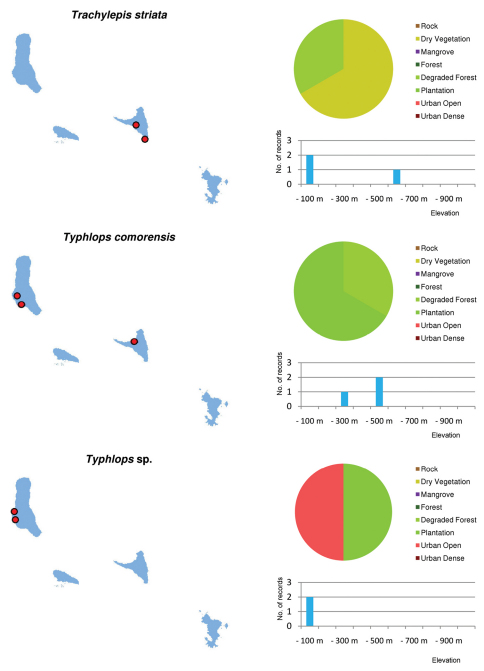
Distribution maps, and distribution over habitat and altitude classes, for *Trachylepis striata*, *Typhlops comorensis* and *Typhlops* sp.

### Areas of conservational importance and future measures

**Figure 20.**
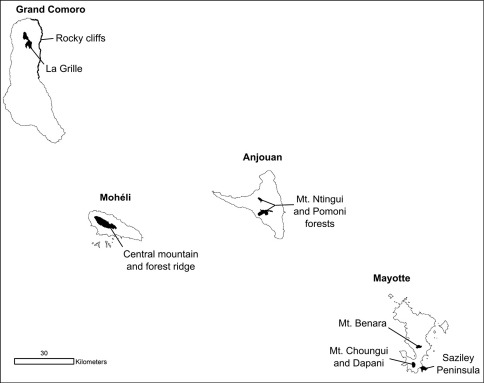
Proposed areas of conservational importance for the herpetofauna on the Comoro islands.

In this section, we propose seven areas of highest importance for the conservation of the Comorian herpetofauna. Our proposals are based on the distributions of native, threatened species, on the occurrence of natural vegetation types and on the degree of potential threats. Figure 20 shows these proposed areas.

Studies concerning α- and β-diversity of birds and butterflies on Anjouan have been undertaken by the ECDD (Engagement Communautaire pour le Developpement Durable, BP 279, Mutsamudu, Anjouan, Comoros; Marsh et al. 2010) that highlight areas of conservational importance for these groups. As stated in this paper, “using just one taxon when prioritising areas for conservation may lead to a poor representation of wider diversity”. Therefore, we underline that in order to benefit the Comorian fauna as a whole, further taxa and factors must be taken into consideration for the planning of protected areas.

### Rocky cliffs along the north-eastern coast of Grand Comoro

This area encompasses an almost linear habitat of coastal cliffs of volcanic rocks. It stretches over a length of about 38 km from the area of Ivouani southwards to Itsikoudi. This is the only habitat of the Comoro iguana *Oplurus cuvieri comorensis*. This taxon inhabits the mostly vertical cliff structures, but also neighbouring areas of volcanic rock which extend several hundreds of meters inland. The only other reptile species detected in this habitat were *Cryptoblepharus boutonii* and *Trachylepis comorensis*. Since the cliff structures do not border the coastline continuously, *Oplurus cuvieri comorensis* cannot be observed along the entire area of the coast. Instead, the cliff areas are home to subpopulations divided by unsuitable habitats.

Further studies should be undertaken to estimate the population size of *Oplurus cuvieri comorensis*, to detect all of its subpopulations and to infer the degree of separation of these subpopulations. Additionally, the development of the agama population on Grand Comoro should be closely monitored. *Oplurus cuvieri comorensis* is currently protected by Comorian law. Nevertheless, efforts should be made to ensure the effectiveness of this protection by preventing the regular capture of individuals by locals for food, entertainment or for the international pet trade. This could be achieved by communication with authorities in the local village communities and particularly by the participation of locals in the works mentioned above.

### La Grille (Grand Comoro)

Grand Comoro is an island of two volcanoes, the northern, older, and lower of which is called La Grille. Aside from several reptile species widespread on Grand Comoro, La Grille is home to the microendemic *Phelsuma comorensis* and probably to the largest population of *Paroedura sanctijohannis* on Grand Comoro. Further endemic species present at La Grille are *Furcifer cephalolepis*, which is very common in this area, and *Lycodryas sanctijohannis*. Forest, of a total area of 747 hectares, remains only in the highest elevations. The mountain is populated up to areas of around 600 m, but plantations and degraded forests with understory crop planting are common up to near the peaks. While *Phelsuma comorensis* mainly inhabits plantations and urban areas and seems not immediately threatened, *Phelsuma sanctijohannis* is found only in or near the forest. Increasing deforestation pressure by a growing population might thus lead to the loss of what to our current knowledge forms the core habitat of the Grand Comoro population of this species.

Efforts should be undertaken to halt the deforestation on La Grille. The populations of *Phelsuma comorensis* and *Phelsuma sanctijohannis* should be monitored, especially as *Phelsuma comorensis* might be exposed to threats by international pet trade and the introduction of further invasive species.

### Central mountain and forest ridge on Mohéli

The largest continuous area of natural and near-natural forest on the Comoros is the forest covering the slopes of Mt. Karthala, Grand Comoro. However, these forests mainly correspond to altitudes of over 1000 m asl and are thus hardly suitable for most reptile species. The second largest forest area is the forest growing on the central mountain ridge of Mohéli. This forest extends from altitudes of around 500 m asl to the peak at 790 m, encompassing an area of about 3190 hectares. It is home to the native reptile species *Paroedura sanctijohannis*, *Phelsuma v-nigra* and *Lycodryas sanctijohannis*.

The forest on Mohéli’s mountain ridge should be considered one of the most important forest areas for conservation on the Comoros not only due to its biodiversity, but also due to its still relatively untouched state. Since Mohéli is more sparsely populated than the other islands of the archipelago, the future rate of deforestation is also expected to be lower. Measures to protect this forest and to halt logging and underplanting would benefit not only reptiles, but also many other groups of organisms (Louette et al. 2004).

### Forests on Anjouan: Mt. Ntingui, Pomoni

Anjouan is the island of the Comoro archipelago most densely populated. Forest remains in higher altitudes and in inaccessible areas. Large parts of the forested areas are in elevations too high for reptiles. In lower altitudes, remarkable forests can be found at the north-western slope of Mt. Ntingui, on the way from Mutsamudu, and above Pomoni. There, pristine forests already grow around 600 m asl. *Paroedura sanctijohannis* is relatively common there. Though not considered endemic, *Geckolepis maculata*, a species rather rare on the Comoros, is regularly observed above Pomoni. Further endemics are *Lycodryas sanctijohannis* and *Phelsuma v-nigra*. The forest at Ntingui, up to about 1000 m asl, covers an area of about 198 hectares, at Pomoni about 940 hectares.

It is currently unknown if the populations of *Phelsuma sanctijohannis* at Mt. Ntingui and Pomoni are separated or if there is gene flow between them. This gene flow might be facilitated either by a more or less closed corridor of suitable habitat between these areas or by a sufficiently large number of wandering individuals (which have also been observed in degraded forest areas). To protect *Phelsuma sanctijohannis* and *Geckolepis maculata* on Anjouan, measures should be taken to protect these two forest areas.

### Mt. Benara (Mayotte)

As also stated in Pascal (2002), the forest at Mt. Benara is one of the best conserved on Mayotte. Two of the three endemic species of day geckos, *Phelsuma nigristriata* and *Phelsuma pasteuri*, are very common there. Mt. Benara is also the only high-altitude forest on Mayotte in which *Paroedura sanctijohannis* has been observed and the top of this mountain is one of only two known locality records for the endemic species *Liophidium mayottensis*. The chameleon *Furcifer cephalolepis*, the two endemic amphibians *Boophis* sp. and *Blommersia* sp. and the non-endemic but rare *Ebenavia inunguis* are also present at Mt. Benara. The forest area is about 185 hectares.

Mt. Benara is already a forest protection area, and on Mayotte, all endemic reptile species are protected. Nevertheless, the importance of Mt. Benara for reptile protection should be emphasized.

### Mt. Choungui and Dapani (Mayotte)

Due to its isolation in the drier southern part of Mayotte, the forest at Mt. Choungui and Dapani is distinguished from other forests of similar altitudes on Mayotte by its vegetation and fauna (Pascal 2002). Most conspicuous among the reptiles is the endemic *Phelsuma robertmertensi*, otherwise a species of coastal habitats. There are also populations of *Paroedura sanctijohannis* and *Furcifer cephalolepis*. This forest encompasses around 233 hectares. Similarly to Mt. Benara, Mt. Choungui is a protected area.

### Dry forest and bush on the Saziley peninsula (Mayotte)

In our classification, the vegetation at Saziley was assigned the habitat class “dry / low vegetation”. This is because, in contrast to forest of the habitat class “closed forest”, this vegetation consists of low trees and bushes which even in their natural state form a canopy only partly continuous and less dense than humid forests do. Saziley is the most remarkable habitat of *Paroedura sanctijohannis*, being the only place where this species has been recorded at sea level and in a dry area. It is furthermore remarkable because a population of *Paroedura sanctijohannis* exists in syntopy with the invasive *Hemidactylus platycephalus*. The non-endemic but rare *Geckolepis maculata* also occurs.

Saziley is not a forest protection area, but is protected and closely monitored because of its beaches, which are breeding sites of marine turtles. As in the cases of Mt. Benara and Mt. Choungui, the importance of Saziley peninsula for reptile protection should be emphasized. Saziley is a peninsula of about 593 hectares.

## Conclusions

On the Comoro islands, only fragments of the original forest vegetation are left, mostly at high altitudes. However, reptiles and amphibians are more common and species-rich in non-natural habitats, especially plantations, and urban areas are home to a remarkable diversity. This can in part be attributed to the high number of invasive, anthropophilous species. However, many native species are recorded commonly outside the natural forests. *Paroedura sanctijohannis* and *Phelsuma nigristriata* appear to be the only reptiles for which pristine forest is of high importance as habitat. As most species show little dependency on natural habitats, loss of these habitats alone could be identified as a strong threat factor only for the two forest species. The most important threat factor at present is invasive species, primarily among the genera *Hemidactylus*, *Phelsuma* and *Agama*, and mammals. Therefore, habitat degradation and destruction remain an important threat factor, as it creates more suitable conditions for invasive species.

The use of satellite imagery, ground truthing, and species distribution maps allowed us to reliably estimate habitat availability and thus Area of Occupancy according to the criteria of IUCN (2001a). According to our evaluation, three endemic species are proposed for the status Endangered, one for Vulnerable, six for Near Threatened, and two for Data Deficient. Four species are proposed for the status Least Concern. The single most threatened taxon, though endemic only as a subspecies, is the Comoro iguana *Oplurus cuvieri comorensis*.

In comparison to Madagascar, where conservation is in the focus of international attention (Kremen et al. 2008), the Comoros are in urgent need of plans and measures for conservation of their native biodiversity. Legislation already provides nominal protection for the Comorian species on the international (CITES regulations for genera *Furcifer* and *Phelsuma*) and national level (see Anonymus 2000, Louette et al. 2004). However, any measures can be effective only if the local communities are conscious of the value of biodiversity and its fragility, and they participate in translating these measures into action.

## Appendix I

List of locality data used in the analyses. (doi: 10.3897/zookeys.144.1648.app1). File format: Microsoft Excel (XLS).

**Explanation note:** List of locality data used in the analyses. Altitude level is given in hundred meters asl (e.g., 4 = 300 to 400 m asl). Abbreviations: APO = Authors’ personal observation (including reports by locals), MCPO = M. Carretero personal observation, see Carretero et al. (2005). SOH (tissue samples, Oliver Hawlitschek personal collection) numbers, like ZSM numbers, are stored at ZSM.

## Appendix II

List of specimens examined. (doi: 10.3897/zookeys.144.1648.app2). File format: Microsoft Excel (XLS).

**Explanation note:** List of specimens examined. Museum abbreviations are explained under “Locality data and species identification” in the “Methods” section.

## References

[B1] Allibert C Argant A Argant J (1983) La site de Dembeni. Etudes Ocean Indien 2: 127-142.

[B2] Allibert C Vérin P (1996) The early pre-islamic history of the Comores islands: links with Madagascar and Africa. In: Reade J (Ed). The Indian Ocean in Antiquity. London, New York: Kegan Paul International, British Museum: 461-470.

[B3] Anonymus (2000) Stratégie nationale et plan d’action pour la conservation de la diversité biologique en RFI des Comores. République Fédérale Islamique des Comores, Ministère de la Production et de l’Environnement, Direction Générale de l’Environnement, 162 pp.

[B4] Atkinson IAW (1985) The spread of commensal species of *Rattus* to oceanic islands and their effects on island avifaunas. In: Moors PJ (Ed). Conservation of island birds. International Council for Bird Preservation, Cambridge: 35-81.

[B5] Bachélery P Coudray J (1993) Carte géologique des Comores. Notice explicative de la carte volcano-tectonique de la Grande Comore (Ngazidja). Ministère Français de la Coopération (Mission Français de Coopération à Moroni – Comores).

[B6] Battistini R Vérin P (1984) Géographie des Comores. Agence de Cooperation culturelle et technique, Editions Fernand Nathan, Paris. 142 pp.

[B7] Bauer AM Jackman TR Greenbaum E de Silva A Giri VB Das I (2010) Molecular evidence for the taxonomic status of *Hemidactylus brookii* group taxa (Squamata: Gekkonidae). Herpetological Journal 20: 129-138.

[B8] Bell KE Donnelly MA (2006) Influence of forest fragmentation on community structure of frogs and lizards in Northeastern Costa Rica. Conservation Biology 20: 1750-1760. 10.1111/j.1523-1739.2006.00522.x 17181810

[B9] Benson CW (1960) The birds of the Comoro islands: results of the British Ornithologists’ Union Centenary expedition 1958. Ibis 103b: 5–106. 10.1111/j.1474-919X.1960.tb03677.x

[B10] Blanc CP (1971) Les reptiles de Madagascar et les îles voisines. Annales de la Université de Madagascar 8: 95-178.

[B11] Blanc CP (1972) Les reptiles de Madagascar et des îles voisines. In: Battistini R, Richard-Vindard G (Eds) Biogeography and Ecology in Madagascar, 501–614.

[B12] Brygoo ER (1981) Systématique des lézards Scincidés de la région malgache. VIII. Les Mabuya des îles de l’Océan Indien occidental: Comores, Europa, Seychelles. Bulletin du Muséum national d’Histoire naturelle 3: 911-930.

[B13] Buchanan GM Butchart SHM Dutson G Pilgrim JD Steininger MK Bishop KD Mayaux P (2008) Using remote sensing to inform conservation status assessment: Estimates of recent deforestation rates on New Britain and the impacts upon endemic birds. Biological Conservation 141: 56-66. 10.1016/j.biocon.2007.08.023

[B14] Burton J (2003) On Red Lists and IUCN. PlantTalk 32: 4-5.

[B15] Carpenter AI (2009)*Furcifer cephalolepis*. In: IUCN 2011. IUCN Red List of Threatened Species. Version 2011.1. www.iucnredlist.org. Downloaded on 05 October 2011.

[B16] Carretero MA Harris DJ Rocha S (2005) Recent observations of reptiles in the Comoro Islands (Western Indian Ocean). Herpetological Bulletin 91: 19-28.

[B17] Case TJ Bolger T Richman AD (1992) Reptilian extinctions: the last ten thousand years. In: Fiedler PL Jain SK (Eds). Conservation biology: the theory and practice of nature conservation, preservation and management. Chapman and Hall, New York: 90-125.

[B18] Central Intelligence Agency (2009) The World Factbook 2009. Washington, DC. https://www.cia.gov/library/publications/the-world-factbook/index.html

[B19] Cheke A (2010) The timing of arrival of humans and their commensal animals on Western Indian Ocean oceanic islands. Phelsuma 18: 38-69.

[B20] Cheke A Hume J (2008) Lost Land of the Dodo. T & AD Poyser, London, 480 pp.

[B21] Chen J Saunders SC Crow TR Naiman RJ Brosofske KD Mroz GD Brookshire BL Franklin JF (1999) Microclimate in forest ecosystem and landscape ecology? Variations in local climate can be used to monitor and compare the effects of different management regimes. BioScience 49: 288-297. 10.2307/1313612

[B22] Chuang SA Lee LL (1997) Food habits of three carnivore species (*Viverricula indica*, *Herpestes uwa*, and *Melogale moschata*) in Fushan Forest, northern Taiwan. Journal of Zoology 243: 71–79. doi; 10.1111/j.1469-7998.1997.tb05757.x

[B23] Cole C (1992) Reptiles and amphibians of the Comoro archipelago. In: Trewhalla WJ Reason PF (Eds). The final report of the University of Bristol Comoros ‘92 expedition. Action Comores. The University of Bristol: 113-120.

[B24] Cole NC Jones CG Harris S (2005) The need for enemy-free space: the impact of an invasive gecko on island endemics. Biological Conservation 125: 467-474. 10.1016/j.biocon.2005.04.017

[B25] Colonna M Casanova J Dullo WC Camoin G (1996) Sea level changes and δ^18^O record for the past 34,000 yr from Mayotte reef, Indian Ocean. Quaternary Research 46: 335-339.

[B26] Daszak P Cunninham AA Hyatt AD (2003) Infectious disease and amphibian population declines. Diversity and Distributions 9: 141-150. 10.1046/j.1472-4642.2003.00016.x

[B27] Denny CM Morley CG Chadderton WL Hero JM (2005) Demonstration project to eradicate invasive cane toads and mammals from Viwa Island, Fiji. Unpublished project plan at the Cooperative Islands Initiative, University of Auckland, 15 pp.

[B28] Diamond JC (1984a) Historic extinctions: their mechanisms, and lessons for understanding prehistoric extinctions. In: Martin PS Klein R (Eds). Quartenary extinctions. University of Arizona Press, Tucson: 824-862.

[B29] Diamond JC (1984b) “Normal” extinctions of isolated populations. In: Nitecki MH (Ed). Extinctions. University of Chicago Press, Chicago: 191-246.

[B30] Emerick CM Duncan RA (1982) Age progressive volcanism in the Comoros archipelago, western Indian Ocean and implication for Somali plate tectonics. Earth and Planetary Science Letters 60: 415-428. 10.1016/0012-821X(82)90077-2

[B31] Fredericksen NJ Fredericksen T S (2002) Terrestrial wildlife responses to logging and fire in a Bolivian tropical humid forest. Biodiversity and Conservation 11: 27-38 10.1023/A:1014065510554

[B32] Gerlach J Canning KL (2004) Evolution and history of the giant tortoises of the Aldabra Island Group. Phelsumania, http://www.phelsumania.com/public/articles/fauna_dipsochelys_1.html

[B33] Glaw F Vences M (2007) A fieldguide to the amphibians and reptiles of Madagascar. Third edition. Vences & Glaw Verlag, Cologne, 496 pp.

[B34] Günther A (1879) On mammals and reptiles from Johanna, Comoro Islands. Annals and Magazine of Natural History 5: 215-218

[B35] Hajash A Armstrong RL (1972) Paleomagnetic and radiometric evidence for the ages of the Comoro islands, west central Indian Ocean. Earth and Planetary Science Letters 16: 231-236. 10.1016/0012-821X(72)90195-1

[B36] Hawlitschek O (2008) Reptiles and Amphibians of the Comoro Islands. Unpublished Diploma Thesis, University of Munich, 257 pp.

[B37] Hayes D Sader SA (2001) Change detection techniques for monitoring forest clearing and regrowth in a tropical moist forest. Photogrammetric Engineering and Remote Sensing 67: 1064-1075.

[B38] Hays WST Conant S (2007) Biology and Impacts of Pacific Island Invasive Species. 1. A Worldwide Review of Effects of the Small Indian Mongoose, *Herpestes javanicus* (Carnivora: Herpestidae). Pacific Science 61: 3-16. 10.1353/psc.2007.0006

[B39] Hoffmann M Hilton-Taylor C Angulo A Böhm M Brooks TM Butchart SH Carpenter KE Chanson J Collen B Cox NA Darwall WR Dulvy NK Harrison LR Katariya V Pollock CM Quader S Richman NI Rodrigues AS Tognelli MF Vié JC Aguiar JM Allen DJ Allen GR Amori G Ananjeva NB Andreone F Andrew P Aquino Ortiz AL Baillie JE Baldi R Bell BD Biju SD Bird JP Black-Decima P Blanc JJ Bolaños F Bolivar-G W Burfield IJ Burton JA Capper DR Castro F Catullo G Cavanagh RD Channing A Chao NL Chenery AM Chiozza F Clausnitzer V Collar NJ Collett LC Collette BB Cortez Fernandez CF Craig MT Crosby MJ Cumberlidge N Cuttelod A Derocher AE Diesmos AC Donaldson JS Duckworth JW Dutson G Dutta SK Emslie RH Farjon A Fowler S Freyhof J Garshelis DL Gerlach J Gower DJ Grant TD Hammerson GA Harris RB Heaney LR Hedges SB Hero JM Hughes B Hussain SA Icochea M J Inger RF Ishii N Iskandar DT Jenkins RK Kaneko Y Kottelat M Kovacs KM Kuzmin SL La Marca E Lamoreux JF Lau MW Lavilla EO Leus K Lewison RL Lichtenstein G Livingstone SR Lukoschek V Mallon DP McGowan PJ McIvor A Moehlman PD Molur S Muñoz Alonso A Musick JA Nowell K Nussbaum RA Olech W Orlov NL Papenfuss TJ Parra-Olea G Perrin WF Polidoro BA Pourkazemi M Racey PA Ragle JS Ram M Rathbun G Reynolds RP Rhodin AG Richards SJ Rodríguez LO Ron SR Rondinini C Rylands AB Sadovy de Mitcheson Y Sanciangco JC Sanders KL Santos-Barrera G Schipper J Self-Sullivan C Shi Y Shoemaker A Short FT Sillero-Zubiri C Silvano DL Smith KG Smith AT Snoeks J Stattersfield AJ Symes AJ Taber AB Talukdar BK Temple HJ Timmins R Tobias JA Tsytsulina K Tweddle D Ubeda C Valenti SV van Dijk PP Veiga LM Veloso A Wege DC Wilkinson M Williamson EA Xie F Young BE Akçakaya HR Bennun L Blackburn TM Boitani L Dublin HT da Fonseca GA Gascon C Lacher TE Jr Mace GM Mainka SA McNeely JA Mittermeier RA Reid GM Rodriguez JP Rosenberg AA Samways MJ Smart J Stein BA Stuart SN (2010) The Impact of Conservation on the Status of the World’s Vertebrates. Science 330: 1503-1509. 10.1126/science.1194442 20978281

[B40] Horner P (2007) Systematics of the snake-eyed skinks, *Cryptoblepharus* Wiegmann (Reptilia: Squamata: Scincidae) – an Australian based review. The Beagle Supplement 3: 21-198.

[B41] Ineich I (2009a)*Cryptoblepharus ater*. In: IUCN 2011. IUCN Red List of Threatened Species. Version 2011.1. www.iucnredlist.org Downloaded on 05 October 2011.

[B42] Ineich I (2009b)*Phelsuma comorensis*. In: IUCN 2011. IUCN Red List of Threatened Species. Version 2011.1. www.iucnredlist.org Downloaded on 05 October 2011.

[B43] Ineich I (2009c)*Phelsuma v-nigra*. In: IUCN 2011. IUCN Red List of Threatened Species. Version 2011.1. www.iucnredlist.org Downloaded on 05 October 2011.

[B44] Ineich I Meirte D (2009)*Amphiglossus johannae*. In: IUCN 2011. IUCN Red List of Threatened Species. Version 2011.1. www.iucnredlist.org Downloaded on 05 October 2011.

[B45] INSEE (2007) INSEE Infos 32, Institut National de la Statistique et des Etudes Economiques, Mayotte.

[B46] IUCN (1994) IUCN Red List Categories. IUCN-SSC.

[B47] IUCN (2001a) IUCN red list categories and criteria (version 3.1). IUCN Species Survival Commission. IUCN, Gland, Switzerland and Cambridge, UK.

[B48] IUCN (2001b) IUCN habitat authority file (version 2.1). IUCN, Gland, Switzerland and Cambridge, UK.

[B49] Jennings SB Brown ND Sheil D (1999) Assessing forest canopies and understorey illumination: canopy closure, canopy cover and other measures. Forestry 72: 59-74. 10.1093/forestry/72.1.59

[B50] Jolly R Fukuda-Parr S (2000) Human Development Report 2000. United Nations Development Programme. Oxford University Press, New York, USA.

[B51] Köhler J Glaw F Vences M (1997) First record of *Mabuya comorensis* (Reptilia: Scincidae) for the Madagascan fauna, with notes on the reptile fauna of the offshore island Nosy Tanikely. Museo Regionale di Scienze Naturali, Bolletino (Torino) 15: 75-82.

[B52] Kothari A (2003) Cadre de géstion environmental et social. Rapport provisionel. Republique Fédérale Islamique des Comores, FADC III, 51 pp.

[B53] Kremen C Cameron A Moilanen A Phillips SJ Thomas CD Beentje H Dransfield J Fisher BL Glaw F Good TC Harper GJ Hijmans RJ Lees DC Louis E Jr. Nussbaum RA Raxworthy CJ Razafimpahanana A Schatz GE Vences M Vieites DR Wright PC Zjhra ML (2008) Aligning conservation priorities across taxa in Madagascar with high-resolution planning tools. Science 320: 222-226. 10.1126/science.1155193 18403708

[B54] Lee TM Jetz W (2011) Unravelling the structure of species extinction risk for predictive conservation science. Proceedings of the Royal Society of London B 278: 1329-1338. 10.1098/rspb.2010.1877 PMC3061137 20943690

[B55] Louette M Meirte D Jocqué R (Eds) (2004) La faune terrestre de’l archipel des Comores. Studies in Afrotropical Zoology 293. Tervuren: MRAC, 456 pp.

[B56] Lowe S Browne M Boudjelas S De Poorter M (2000) 100 of the world’s worst invasive alien species. A selection from the Global Invasive Species Database. Published by The Invasive Species Specialist Group (ISSG), a specialist group of the Species Survival Commission (SSC) of the World Conservation Union (IUCN).

[B57] Marsh CJ Lewis OT Said I Ewers RE (2010) Community-level diversity modelling of birds and butterflies on Anjouan, Comoro Islands. Biological Conservation 143: 1364-1374. 10.1016/j.biocon.2010.03.010

[B58] Mausfeld-Lafdhiya P Schmitz A Ineich I Chirio L (2004) Genetic variation in two African *Euprepis* species (Reptilia, Scincidae), based on maximum-likelihood and Bayesian analyses: taxonomic and biogeographic conclusions. Bonner Zoologische Beiträge 52: 159-177.

[B59] Meirte D (1992) Occurrence of *Oplurus cuvieri* (Reptilia, Iguanidae) on Grand Comoro, Indian Ocean. Bulletin of the British Herpetological Society 39: 3-4.

[B60] Meirte D (2004) Reptiles. Batraciens. In: Louette M Meirte D Jocqué R (Eds) (2004) La faune terrestre de’l archipel des Comores. Studies in Afrotropical Zoology 293. Tervuren: MRAC, 199–226.

[B61] Mendelson JR 3rd Lips KR Gagliardo RW Rabb GB Collins JP Diffendorfer JE Daszak P Ibáñez D R Zippel KC Lawson DP Wright KM Stuart SN Gascon C da Silva HR Burrowes PA Joglar RL La Marca E Lötters S du Preez LH Weldon C Hyatt A Rodriguez-Mahecha JV Hunt S Robertson H Lock B Raxworthy CJ Frost DR Lacy RC Alford RA Campbell JA Parra-Olea G Bolaños F Domingo JJ Halliday T Murphy JB Wake MH Coloma LA Kuzmin SL Price MS Howell KM Lau M Pethiyagoda R Boone M Lannoo MJ Blaustein AR Dobson A Griffiths RA Crump ML Wake DB Brodie ED Jr. (2006) Confronting amphibian declines and extinctions. Science 313: 48. 10.1126/science.1128396 16825553

[B62] Montaggioni L Nougier J (1981) Les enclaves de roches détritiques dans les volcans d’Anjouan (Archipel des Comores): origine et interprétation dans le cadre de l’évolution du Canal de Mozambique. Bulletin de la Societé Geologique de France 23: 596-601.

[B63] Msaidie S Ducourneau A Boetsch G Longepied G Papa K Allibert C Yahaya AA Chiaroni J Mitchell MJ (2011) Genetic diversity on the Comoros Islands shows early seafaring as major determinant of human biocultural evolution in the Western Indian Ocean. European Journal of Human Genetics 19: 89-94. 10.1038/ejhg.2010.128 20700146 PMC3039498

[B64] Münchenberg T Wollenberg KC Glaw F Vences M (2008) Molecular phylogeny and geographic variation of Malagasy iguanas (*Oplurus* and *Chalarodon*). Amphibia-Reptilia 29: 319-327. 10.1163/156853808785112101

[B65] Nougier J Chantagrel JM Karche JP (1986) The Comores archipelago in the western Indian Ocean: volcanology, geochronology and geodynamic setting. Journal of African Earth Sciences 5: 135-145. 10.1016/0899-5362(86)90003-5

[B66] Paris B (1999) Espéces de faune et flore connues en RFI des Comores. Projet de conservation de la biodiversité et developpement durable (PNUD/FEM) Moroni, Comores.

[B67] Pascal O (2002) Plantes et forêts de Mayotte. Patrimoines Naturels, 53, Paris, SPN / IEGB / MNHN, 108 pp.

[B68] Purvis A Gittleman J L Cowlishaw G Mace GM (2000) Predicting extinction risk in declining species. Proceedings of the Royal Society of London B 267: 1947-1952. 10.1098/rspb.2000.1234 PMC1690772 11075706

[B69] Raxworthy CJ Forstner MRJ Nussbaum RA (2002) Chameleon radiation by oceanic dispersal. Nature 415: 784-786. 11845207 10.1038/415784a

[B70] Raxworthy CJ Pearson RG Rabibisoa N Rakotondrazafy AM Ramanamanjato J-B Raselimanana AP Wu S Nussbaum RA Stone DA (2008) Extinction vulnerability of tropical montane endemism from warming and upslope displacement: a preliminary appraisal for the highest massif in Madagascar. Global Change Biology 14: 1703-1720. 10.1111/j.1365-2486.2008.01596.x

[B71] Rocha S Carretero M A Harris D J (2005) Diversity and phylogenetic relationships of *Hemidactylus* geckos from the Comoro islands. Molecular Phylogenetics and Evolution 35: 292-299. 10.1016/j.ympev.2004.11.023 15737599

[B72] Rocha S Carretero MA Vences M Glaw F Harris DJ (2006) Deciphering patterns of transoceanic dispersal: the evolutionary origin and biogeography of coastal lizards (*Cryptoblepharus*) in the Western Indian Ocean region. Journal of Biogeography 33: 13-22. 10.1111/j.1365-2699.2005.01375.x

[B73] Rocha S Vences M Glaw F Posada D Harris DJ (2009a) Multigene phylogeny of Malagasy day geckos of the genus *Phelsuma*. Molecular Phylogenetics and Evolution 52: 530-537. 10.1016/j.ympev.2009.03.032 19362158

[B74] Rocha S Ineich I Harris D J (2009b) Cryptic variation and recent bipolar range expansion within the Stumped-Toed Gecko *Gehyra mutilata* across Indian and Pacific Ocean islands. Contributions to Zoology 78: 1-8.

[B75] Rocha S Carretero MA Harris DJ (2010a) Genetic diversity and phylogenetic relationships of *Mabuya* spp. (Squamata: Scincidae) from western Indian Ocean islands. Amphibia-Reptilia 31: 375-385.

[B76] Rocha S Carretero MA Harris DJ (2010b) On the diversity, colonization patterns and status of *Hemidactylus* spp. (Reptilia: Gekkonidae) from the Western Indian Ocean islands. Herpetological Journal 20: 83-89. 10.1163/156853810791769473

[B77] Rodrigues ASL Pilgrim JD Lamoreux JF Hoffmann M Brooks TM (2006) The value of the IUCN Red List for conservation. Trends in Ecology & Evolution 21: 71-76. 10.1016/j.tree.2005.10.010 16701477

[B78] Rödder D Kielgast J Bielby J Schmidtlein S Bosch J Garner TWJ Veith M Walker S Fisher MC Lötters S (2009) Global Amphibian Extinction Risk Assessment for the Panzootic Chytrid Fungus. Diversity 1: 52-66.

[B79] Rödder D Hawlitschek O Glaw F (2010) Environmental niche plasticity of the endemic gecko *Phelsuma parkeri* Loveridge 1941 from Pemba Island, Tanzania: a case study of extinction risk on flat islands by climate change. Tropical Zoology 23: 35-49. 10.3390/d1010052

[B80] Rösler H Glaw F (2010) Morphologische Variation und Taxonomie von Hemidactylus *brookii* Gray, 1845 und *Hemidactylus angulatus* Hallowell, 1854 sowie phänotypisch ähnlicher Taxa. Spixiana 33: 139-160.

[B81] Sader SA Hayes DJ Coan M Hepinstall JA Soza C (2001) Forest change monitoring of a remote biosphere reserve. International Journal of Remote Sensing 22: 1937-1950.

[B82] Skerratt LF Berger L Speare R Cashins S McDonald KR Phillott AD Hines HB Kenyon N (2007) Spread of chytridiomycosis has caused the rapid global decline and extinction of frogs. EcoHealth 4: 125-134. 10.1007/s10393-007-0093-5

[B83] Stuart SN Chanson JS Cox NA Young BE Rodrigues ASL Fischman DL Waller RW (2004) Status and Trends of Amphibian Declines and Extinctions Worldwide. Science 306: 1783-1786. 10.1126/science.1103538 15486254

[B84] Tassin J Rivière J-N Cazanove M Bruzzese E (2006) Ranking of invasive woody plant species for Management on Réunion Island. Weed Research 46: 388-403. 10.1111/j.1365-3180.2006.00522.x

[B85] Temple S (1985) Why endemic island birds are so vulnerable to extinction. In: Temple S (Ed). Bird conservation 2. University of Wisconsin Press, Madison: 3-6.

[B86] UNEP-WCMC (2011) CITES Trade Database. Available at: http://www.unep-wcmc.org/citestrade/ (Accessed: 2011–09–13).

[B87] Vences M Vieites DR Glaw F Brinkmann H Kosuch J Veith M Meyer A (2003) Multiple overseas dispersal in amphibians. Proceedings of the Royal Society of London B 270: 2435-2442. 10.1098/rspb.2003.2516 PMC1691525 14667332

[B88] Vences M Wanke S Vieites DR Branch WR Glaw F Meyer A (2004) Natural colonization or introduction? Phylogeographical relationships and morphological differentiation of house geckos (*Hemidactylus*) from Madagascar. Biological Journal of the Linnean Society 83: 115-130 10.1111/j.1095-8312.2004.00370.x

[B89] Vié JC Hilton-Taylor C Pollock C Ragle J Smart J Stuart S Tong R (2008) The IUCN Red List: a key conservation tool. In: Vié J-C, Hilton-Taylor C, Stuart SN (Eds) The 2008 Review of The IUCN Red List of Threatened Species. IUCN, Gland, Switzerland and Cambridge, UK.

[B90] Wagner P Glaw F Glaw K Böhme W (2009) Studies on African *Agama* IV: First record of *Agama* *agama* (Sauria: Agamidae) from Madagascar and identity of the alien population on Grande Comore Island. Herpetology Notes 2: 73-77.

[B91] Wake DB Vredenburg VT (2008) Are we in the midst of the sixth mass extinction? A review from the world of amphibians. Proceedings of the National Academy of Sciences 105: 11466-11473. 10.1073/pnas.0801921105 PMC2556420 18695221

[B92] Weldon C du Preez LH Hyatt AD Muller R Speare R (2004) Origin of the amphibian chytrid fungus. Emerging Infectious Diseases 10: 2100-2105. 15663845 10.3201/eid1012.030804 PMC3323396

[B93] Wilson EH Sader SA (2002) Detection of forest harvest type using multiple dates of Landsat TM imagery. Remote Sensing of Environment 80: 385-396. 10.1016/S0034-4257(01)00318-2

[B94] Witmer G Burke PW Pitt WC Avery ML (2007) Management of invasive vertebrates in the USA: an overview. In: Witmer GW, Pitt WC, Fagerstone KA (Eds) Managing Vertebrate Invasive Species: Proceedings of an International Symposium. USDA/APHIS/WS, National Wildlife Research Center, Fort Collins, CO, 12 pp.

